# Decay in survival motor neuron and plastin 3 levels during differentiation of iPSC-derived human motor neurons

**DOI:** 10.1038/srep11696

**Published:** 2015-06-26

**Authors:** María G Boza-Morán, Rebeca Martínez-Hernández, Sara Bernal, Klaus Wanisch, Eva Also-Rallo, Anita Le Heron, Laura Alías, Cécile Denis, Mathilde Girard, Jiing-Kuan Yee, Eduardo F. Tizzano, Rafael J Yáñez-Muñoz

**Affiliations:** 1 School of Biological Sciences, Royal Holloway, University of London, Egham, TW20 0EX, UK; 2 Department of Genetics and CIBERER U-705, Hospital de la Santa Creu i Sant Pau, Barcelona, Spain; 3 Institute for Stem Cell Therapy and Exploration of Monogenic Diseases, Evry Cedex, France; 4 Department of Virology, Beckman Research Institute, City of Hope, Duarte, California, USA

## Abstract

Spinal muscular atrophy (SMA) is a neuromuscular disease caused by mutations in *Survival Motor Neuron 1* (*SMN1*), leading to degeneration of alpha motor neurons (MNs) but also affecting other cell types. Induced pluripotent stem cell (iPSC)-derived human MN models from severe SMA patients have shown relevant phenotypes. We have produced and fully characterized iPSCs from members of a discordant consanguineous family with chronic SMA. We differentiated the iPSC clones into ISL-1+/ChAT+ MNs and performed a comparative study during the differentiation process, observing significant differences in neurite length and number between family members. Analyses of samples from wild-type, severe SMA type I and the type IIIa/IV family showed a progressive decay in SMN protein levels during iPSC-MN differentiation, recapitulating previous observations in developmental studies. PLS3 underwent parallel reductions at both the transcriptional and translational levels. The underlying, progressive developmental decay in SMN and PLS3 levels may lead to the increased vulnerability of MNs in SMA disease. Measurements of *SMN* and *PLS3* transcript and protein levels in iPSC-derived MNs show limited value as SMA biomarkers.

SMA is an autosomal recessive neuromuscular disease caused by mutations in the *Survival of Motor Neuron 1* gene (*SMN1*), which result in decreases in SMN protein levels^1^,^2^. Degeneration of alpha motor neurons (MNs) in the ventral horn of the spinal cord, atrophy of skeletal muscles and generalized weakness are hallmarks of the disorder^3^,^4^. SMA is clinically divided in four types broadly grouped by age of onset and highest motor function achieved. In the most severe form (SMA type I), the onset of the disease occurs before 6 months of age and patients usually die within the first two years of life. Type II SMA is the intermediate form; affected patients never walk. Type III disease is characterized by patients who are able to walk; depending on the onset of symptoms, patients can be classified as type IIIa (onset before 3 years) or type IIIb (after 3 years). In SMA type IV, the onset of the pathology occurs within the second or third decade of life and patients only display mild motor impairment^5^,^6^.

Two *SMN* genes are present in the human genome: *SMN1* (telomeric) and *SMN2* (centromeric)^1^. *SMN1* mostly produces full-length mRNA (*FL-SMN*) and functional protein, while *SMN2* mainly produces an alternatively spliced isoform with no exon seven (*Δ7-SMN*), which is thought to encode a less functional and rapidly degraded truncated protein^7^,^8^. Depending on the tissue, about 10–50% of the *SMN2* pre-mRNA can be properly spliced and produce functional SMN^9^,^10^. SMN is ubiquitously produced and the specific susceptibility of MNs is not understood^2^. Observations in SMA patients and mouse models demonstrate the involvement of non-neural tissues in the disease^11^. It has been proposed that all cells are in a vulnerability-resistance spectrum in regards to SMN reduction, MNs being located at the most sensitive end^12^.

The number of *SMN2* copies is the most important known phenotypic modifier of the disease, since a higher number of *SMN2* copies correlates with higher production of properly spliced *SMN* mRNA and thus functional SMN^13^,^14^, but this correlation is not always absolute^15^. Siblings with the same *SMN1-SMN2* genotype but discordant phenotype have been described in SMA families^16^,^17^,^18^,^19^,^20^,^21^. High levels of plastin 3 (PLS3) in lymphoblasts and blood have been associated with a positive modifier effect on SMA severity in discordant female siblings^22^,^23^,^24^, but there seem to be additional unknown modifier factors regulating the severity of the disease^25^. We have worked extensively with an SMA family in which four discordant sisters born from consanguineous parents are homozygous for a 4-bp deletion in *SMN1* exon 3 (c.399_402delAGAG). This mutation produces a frame-shift that predicts a stop codon 40-bp downstream^21^,^26^. Despite presenting the same *SMN1* mutation and identical *SMN2* copy number (*n* = 4), the clinical manifestations in the four sisters range from early onset SMA type IIIa to SMA type IV with minimal manifestations.

The recent derivation and further differentiation of iPSCs from patients with type I SMA has provided new models to study the disease and shown MN phenotypes in cell culture^27^,^28^,^29^,^30^. Here we report the production and differentiation of iPSC lines derived from members of the c.399_402delAGAG family, who present with less severe phenotypes. Differentiation of these novel iPSCs towards the MN lineage showed mild neurite defects in the affected sisters compared to the carrier mother but no obvious effect in neuroepithelial-derived spheres (Nsphs)-myotube co-cultures. We show a progressive decline of SMN and PLS3 levels during the iPSC to MN differentiation, confirmed in wild-type and unrelated type I SMA iPSCs. Finally, we discuss the limitations of *SMN* and *PLS3* transcript and protein levels as SMA biomarkers, even when the measurements are obtained from iPSC-derived MN cultures.

## Results

### Generation and characterisation of iPSCs

**Figure 1:**
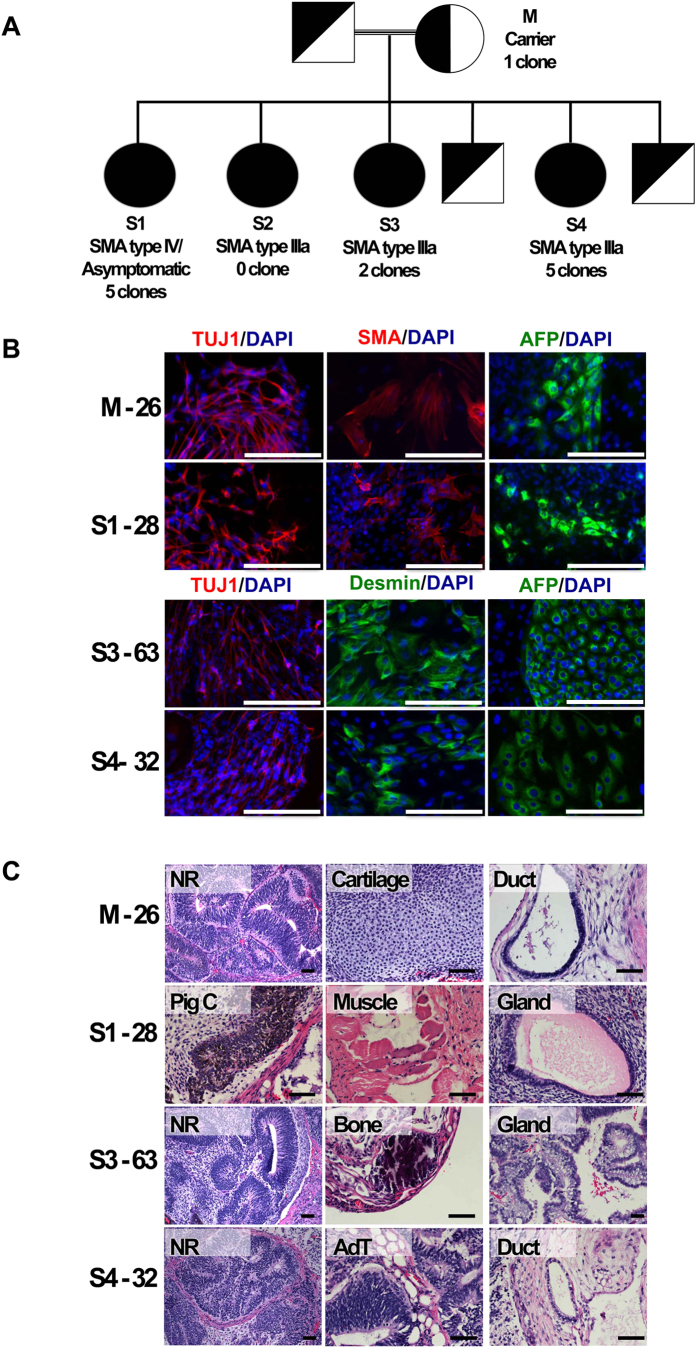
Pedigree chart, iPSC clones obtained and pluripotency assessment. (**A**) Diagram showing the carrier parents and siblings (black and white symbols) and the SMA type IV/asymptomatic and SMA type IIIa siblings (black circles) harbouring the *SMN1* mutation (c.399_402delAGAG, exon 3). For each individual, code, phenotype and number of iPSC clones generated are shown. (**B**) *In vitro* differentiation potential of iPSC clones. EBs produced from selected iPSC clones from each individual generated cell derivatives of the three primary germ cell layers. Immunofluorescence analysis shows the expression of markers from ectoderm (TUJ1, red), mesoderm (smooth muscle actin, SMA, red; desmin, green) and endoderm (AFP, green). Nuclei were counterstained with DAPI. Scale bar = 200 μm. Other abbreviations: TUJ1, neuronal class III beta tubulin; AFP, alpha fetoprotein; DAPI, 4’,6-diamidino-2-phenylindole. (**C**) *In vivo* differentiation potential of iPSC clones. Two million cells from selected iPSC clones were injected into Fox Chase SCID-beige mice and teratomas were obtained 36 days later. Hematoxylin and eosin staining shows the presence of pigmented cells (PigC) and neural rosettes (NR) of ectodermal origin; muscle, bone, cartilage and adipose tissue (AdT) of mesodermal origin; glands and duct of endodermal origin. Scale bar = 100 μm.

We generated several iPSC clones from skin fibroblasts from four family members (M, S1, S3, S4) through retroviral transduction with Yamanaka’s set of reprogramming factors^31^ (Fig. 1A). One clone from each individual was selected and further propagated and characterised, rendering four stable iPSC lines: M-26, S1-28, S3-63 and S4-32. All selected clones showed successful reprogramming as judged by the expression of pluripotency markers (Supplementary Fig. S1 and S2), down-regulation of exogenous transgenes and up-regulation of their endogenous expression (Supplementary Fig. S3) and *in vitro* and *in vivo* differentiation potential (Fig. 1B,C). Furthermore, DNA fingerprinting confirmed the identity of the clones and karyotype analysis showed no obvious chromosomal aberration (Supplementary Table S1 and Fig. S4).

### iPSC-MN differentiation and patient-specific differences in neurite outgrowth

**Figure 2:**
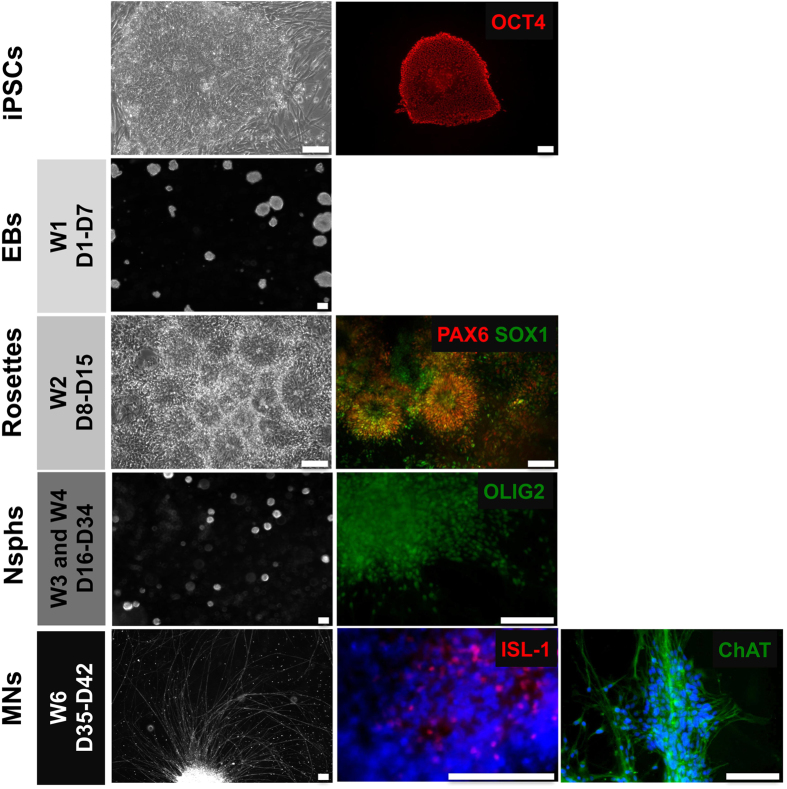
iPSC-MN differentiation process. Representative samples were analysed during the differentiation process and the identities of specific cell types were confirmed by immunocytochemistry: OCT4+ iPSCs at D0, EBs at D7, PAX6+/SOX1+ rosettes at D15, OLIG2+ differentiating Nsphs containing MN progenitors at D28, ISL−1+ MNs at D35 and ChAT+ MNs at D42. Scale bar: 100 μm. Abbreviations: EBs, embryoid bodies; Nsphs, differentiating neuroepithelial-derived spheres; W, week; OCT4, POU class 5 homeobox 1; PAX6, Paired box gene 6; SOX1, Sex determining region Y-box 1; OLIG2, Oligodendrocyte 2; ChAT, choline acetyl transferase.

We differentiated the four iPSC lines from the family under study into MNs following an embryoid body (EB)-based differentiation protocol for human embryonic stem cells (hESCs)^32^ (Fig. 2). iPSCs were cultured in suspension as EBs for seven days to induce loss of pluripotency and initial differentiation into a neuronal fate. Twelve to fourteen days (12-14D) after the start of the differentiation protocol PAX6+/SOX1+ neural tube-like rosettes were observed (Supplementary Fig. S5). Rosettes were selected manually and cultured in suspension as differentiating Nsphs for variable lengths of time depending on the experiment. By D28, most of the cells in the Nsphs were olig2+ MN progenitors. Plating of D28 Nsphs and culture in MN-specific conditions for 1–2 weeks (D35-47) induced cell cycle arrest, the appearance of ISL−1+ MNs and the growth of neurites which could reach more than 2 mm in length if big clusters were seeded in close proximity (>500 μM in diameter, ~1 cluster/cm^2^). ChAT expression was seen only after D40. In line with other reports, a semi-quantitative analysis of ISL-1 expression showed approximate MN yields of ~10–15% within the heterogeneous D35 populations derived from the differentiation of the clones (Supplementary Fig. S6). Further confirmation of MN generation and identity was obtained by live fluorescent marking. For this, cells from differentiating D22 Nsphs from clone S3-63 were transduced with a lentiviral reporter vector expressing red fluorescent protein (RFP) under the MN-specific HB-9 enhancer/promoter^33^,^34^,^35^. RFP expression could already be seen at D24, and the proportion of RFP+ cells increased strongly after D34, with MN yields similar to those estimated by ISL-1 immunochemistry (Supplementary Fig. S7). More accurate determination of MN yields was not possible due to the 3D nature of the differentiating Nsphs. A similar proportion of RFP+ cells was still evident at D43, suggesting that most of the active MN production occurred on the 5^th^ week of MN differentiation (D34–D43) as it has been previously described in fixed hESC-derived MN cultures^36^.

**Figure 3:**
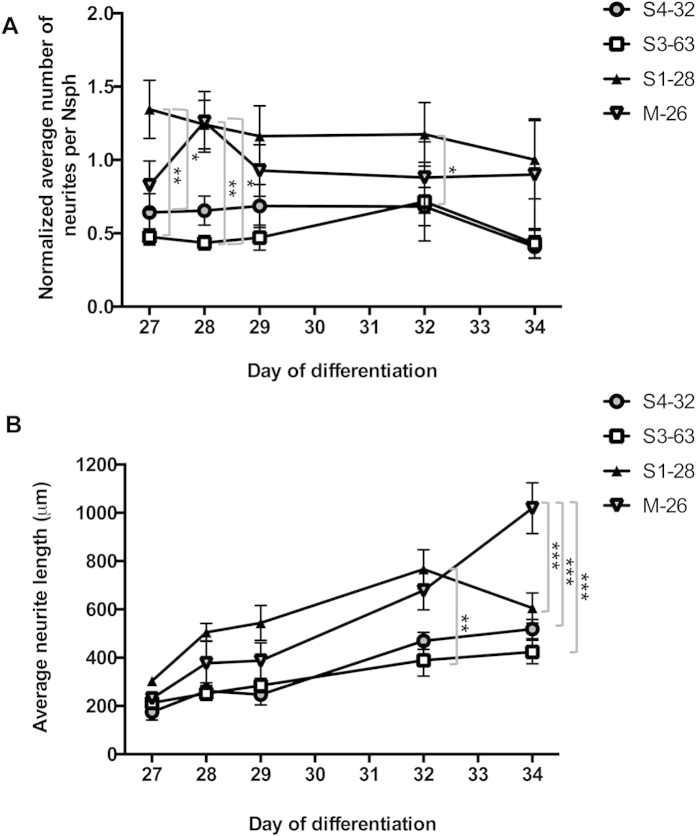
Neurite growth analysis in differentiating iPSC-derived Nsphs containing MN progenitors. Small D26 Nsphs (150–200 μm in diameter) from the indicated iPSC clones were plated in Poly-L-Ornithine-laminin coated dishes for consecutive neurite length measurements over 8 days, as described in Materials and Methods. (**A**) Average number of neurites per Nsph normalised to Nsph size. (**B**) Average neurite length. Data are presented as mean ± SEM. *p < 0.05; **p < 0.01; ***p < 0.001.

The development of iPSCs into MNs is morphologically characterised by radial outgrowth of neurites from the centre of the differentiating Nsphs. In order to investigate patient-specific differences, the average neurite number (normalised by Nsph size) and length for each differentiating Nsph were analysed between D27 and D34. The method we used is illustrated in Supplementary Fig. S8, and the results shown in Fig. 3. Maternal clone M-26 and Type IV clone S1-28 showed trends with higher values than those observed in type III clones. The differences reached statistical significance in some cases, as indicated in Fig. 3.

### Co-culture of MNs and mouse myotubes

**Figure 4:**
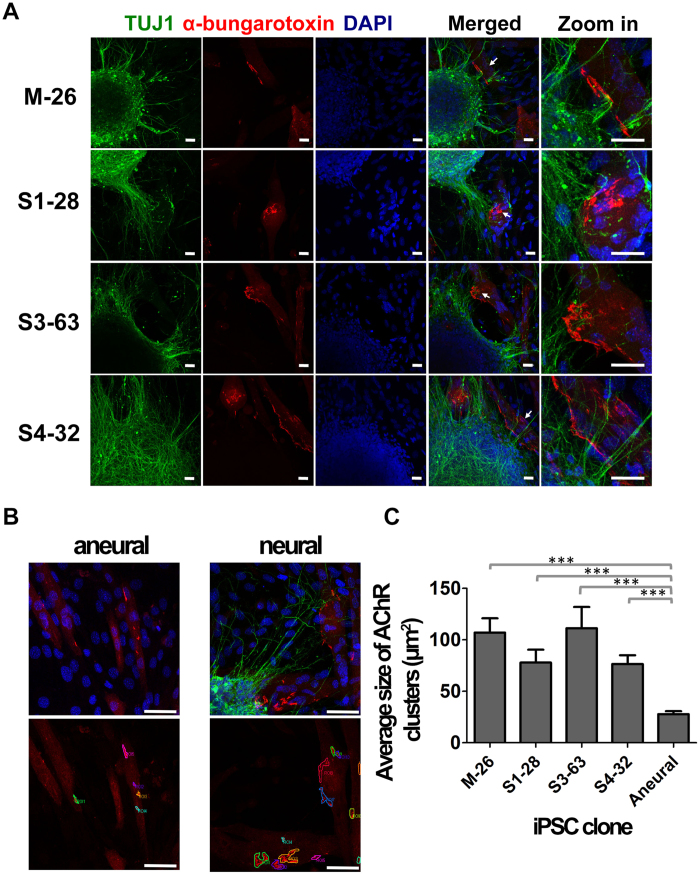
Co-cultures of differentiating Nsphs and C2C12 myoblasts. (**A**) Co-cultures of iPSC derived MNs from the family under study and mouse myotubes. D39 Nsphs from differentiating iPSC clones were seeded onto differentiating C2C12 mouse myoblasts and cultured for 3 days in a mixed MN/muscle medium. Acetylcholine receptors (AChRs), identified by α-bungarotoxin staining (red), clustered preferentially in fibres located around TUJ1+ (green) neurites emerging from the differentiating Nsphs. Confocal imaging suggests that at least some neurite terminals co-localised with AChR plaques. The same pattern was observed in co-cultures of all family clones. Arrows on “Merged” panel point to clusters of AChRs co-localising with neurite terminals, shown at higher magnification on the last column of images. Nuclei were counterstained with DAPI (blue). Scale bar: 20 μm. Abbreviations: TUJ1, neuronal class III beta tubulin; DAPI, 4’,6-diamidino-2-phenylindole. (**B**) Comparison of AChR clustering in aneural myoblast cultures and neural co-cultures. C2C12 mouse myoblasts cultured aneurally show diffuse location of AChRs, with few, small clusters, whereas their co-culture with D39 Nsphs (for 3 days) induces localised expression and higher clustering. The lower images show how AChR clustes were measured by manually demarcating the regions of interest. Scale bar: 50 μm. (**C**) Average size of AChR clusters formed upon iPSC-MN/C2C12 myotube interaction. The quantitation was performed with family clones, using aneural cultures as controls. Data are presented as mean ± SEM. ***p < 0.001.

iPSC-derived differentiating Nsphs were also co-cultured on differentiated mouse C2C12 myoblasts. In all clones it was possible to observe specific co-localisation of TUJ1+ neurites with compact clusters of α-bungarotoxin-stained acetylcholine receptors (AChRs), indicative of neuromuscular junction (NMJ)-like structures (Fig. 4A). Aneural (in the absence of Nsphs) myoblast cultures showed expression of AChRs, but these mostly presented in a few small clusters dispersed along the fibres, without any particular localisation preference (Fig. 4B). Addition of differentiating Nsphs promoted clustering of AChRs in fibres surrounding the Nsphs (Fig. 4A). Quantitative analysis of the average size of the NMJ-like plaques showed significantly smaller structures in aneural cultures compared to the co-cultures, but no statistically significant difference was found between the co-cultures of the family members (Fig. 4C). NMJ-like plaques in aneural cultures and in areas of co-cultures lacking direct contact with differentiating Nsphs were not significantly different in size, ruling out possible effects of factors secreted to the medium by the Nsphs. The presence of these smaller plaques in aneural cultures suggests that spontaneous AChR clustering occurs in myotubes but fails to mature in the absence of direct contact with MN terminals, while differentiating Nsphs from carrier mother and sisters were equally able to induce AChR clustering under these conditions.

### Variation of SMN and PLS3 levels during iPSC-MN differentiation

**Figure 5:**
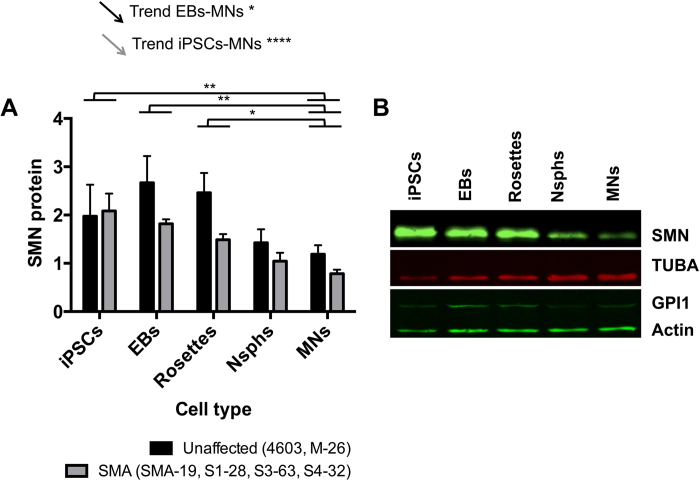
Decay in SMN protein levels during iPSC-MN differentiation. (**A**) SMN protein levels were analysed by western blot at the indicated differentiation stages and samples grouped as unaffected or SMA. Statistical significance for differences between differentiation stages is indicated. No significant differences were found between unaffected and SMA groups at any cell stage. Arrows represent the statistical significance of the linear trend for gradual decrease along the MN differentiation process for each group. Data are presented as mean ± SEM. *p < 0.05; **p < 0.01; ****p < 0.0001. (**B**) Representative western blot showing decay in SMN protein during the iPSC-MN differentiation of clone S3-63. Abbreviations: EBs, embryoid bodies; Nsphs, differentiating neuroepithelial-derived spheres; MNs, motor neurons; TUBA, alpha tubulin; GPI1, glucose phosphate isomerase.

To compare gene expression levels between SMA family members during the iPSC-MN differentiation process, five different cell culture stages were collected, including iPSCs (D0), EBs (D7), rosettes (D15), differentiating Nsphs (D35) and MNs (D42). We also included as a reference the parental fibroblasts from which the iPSC clones were generated. We followed the expression of *SMN* at the transcriptional and translational levels. iPSC clones showed some variations in mRNA expression levels of *FL-SMN* and *Δ7-SMN*, with a bell-shaped distribution in most cases, but fairly constant total *SMN* (*tSMN)* and *FL-SMN* to *Δ7*-*SMN* (*FL/Δ7*-*SMN)* mRNA ratios through the iPSC-MN differentiation stages (Supplementary Fig. S9). In contrast, the clones exhibited a gradual decrease in SMN protein during the MN differentiation process, irrespective of their initial absolute values at the iPSC stage (Supplementary Fig. S9). The only exception to this pattern was found in clone M-26, which had particularly low SMN protein levels at the iPSC stage. To confirm whether the gradual decrease of SMN protein was idiosyncratic to the family or a general characteristic during iPSC-MN differentiation, we performed the same expression analyses in male wild-type and type I SMA iPSCs (4603 and SMA-19 respectively), obtaining similar results (Supplementary Fig. S9). We also analysed SMN protein levels grouping the samples as unaffected (wild-type control 4603 and carrier M-26) and SMA (all types). This confirmed statistically significant linear reductions of SMN protein levels during MN differentiation in both control and SMA groups (Fig. 5).

**Figure 6:**
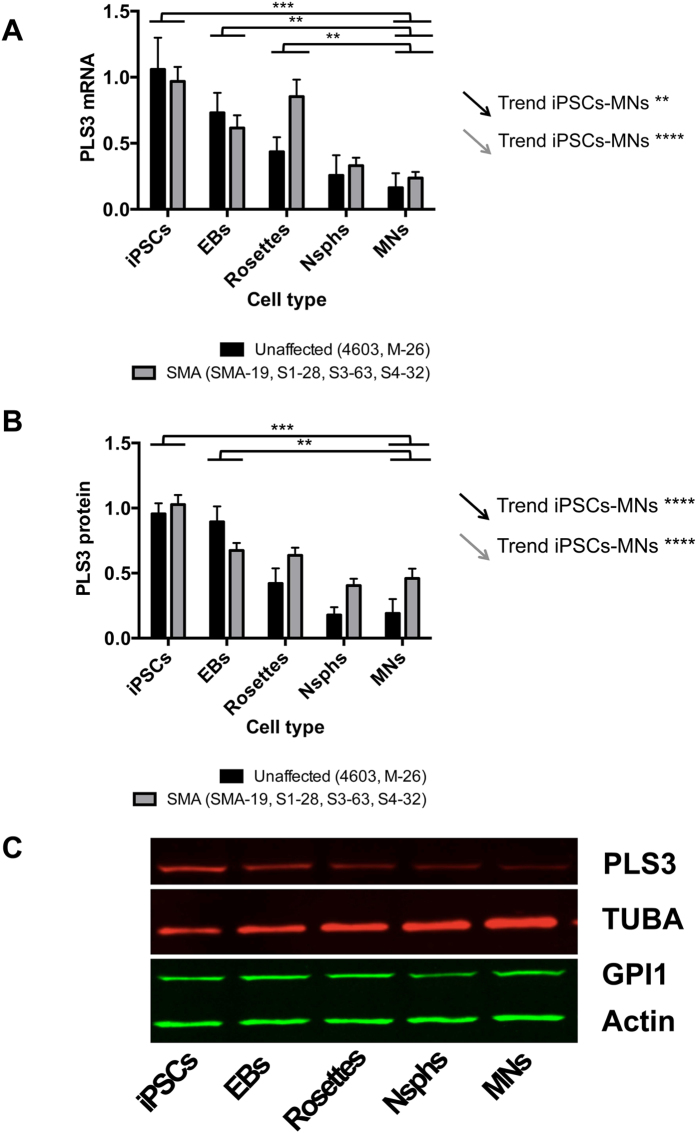
Decay in PLS3 levels during iPSC-MN differentiation. Levels of *PLS3* mRNA (**A**) and protein (**B**) were analysed at the indicated differentiation stages and samples grouped as unaffected or SMA. Arrows represent the statistical significance of the linear trend for gradual decrease along the MN differentiation process for each group. Statistical significance for differences between differentiation stages is also indicated. No significant differences were found between control and SMA groups at any cell stage. Data are presented as mean ± SEM. **p < 0.01; ***p < 0.001; ****p < 0.0001. (**C**) Representative western blot showing decay in PLS3 protein during the iPSC-MN differentiation of clone S3-63. Abbreviations: EBs, embryoid bodies; Nsphs, differentiating neuroepithelial-derived spheres; MNs, motor neurons; TUBA, alpha tubulin; GPI1, glucose phosphate isomerase.

Since PLS3 has been reported as a modifier of the SMA phenotype in females, we also followed its expression at the transcriptional and translational levels during the iPSC-MN differentiation process. Remarkably, wild-type, carrier mother and SMA clones showed a gradual decrease in *PLS3* mRNA and protein levels (Supplementary Fig. S10). When, as in the case of SMN, *PLS3* transcript and protein values were analysed in unaffected and SMA groups, we found statistically significant linear decays along the iPSC-MN differentiation process (Fig. 6). Similar results were obtained when considering only female individuals (see below).

### SMN and PLS3 measurements as biomarkers in SMA pathology

**Figure 7:**
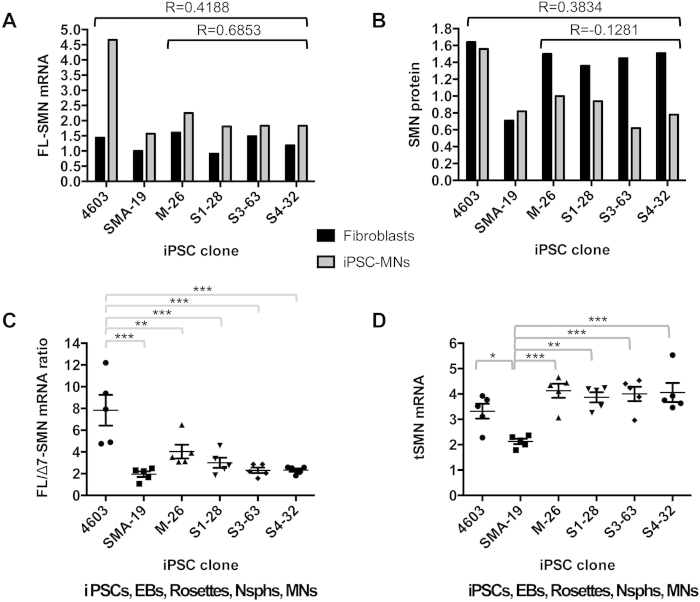
Comparative assessment of SMN measurements. (**A**) The expression of *FL-SMN* mRNA and (**B**) the level of protein in paired fibroblast and their corresponding differentiated MN cultures were compared in all individuals under study; *n* = 2. Parental fibroblasts from iPSC clone SMA-19 were not available and in this case fibroblasts from another type I SMA patient (with two *SMN2* copies and no *SMN1*) were used as an alternative reference. Pearson correlation coefficients (R) for values in fibroblasts and MNs are shown for all individuals combined and for the type IIIa/IV family. Average values for (**C**) *FL/Δ7-SMN* mRNA ratio and (**D**) *tSMN* mRNA from iPSCs, EBs, rosettes, Nsphs and MNs were pooled for each individual for comparison among SMA genotypes and phenotypes. Data are presented as mean ± SEM. *p < 0.05; **p < 0.01; ***p < 0.001. Abbreviations: EBs, embryoid bodies; Nsphs, differentiating neuroepithelial-derived spheres; MNs, motor neurons.

Blood levels of *FL-SMN* mRNA and protein are often assessed in SMA studies, even though their predictive and diagnostic value is limited. We compared measurements of both parameters between the readily available fibroblasts from our subjects of study and their corresponding iPSC-MNs, finding no correlation (Fig. 7A,B). MN values of *FL-SMN* mRNA and protein were higher in the control clone 4603 than in the SMA carrier M-26 and all SMA clones; there was little difference in those MN values between carrier mother and SMA siblings in the discordant family. We assessed possible alterations in *SMN* transcription patterns due to epigenetic changes during fibroblast reprograming and iPSC-MN differentiation through *Dde*I restriction analysis of exons 6–8 of the *SMN* transcripts (*SMN1* and *SMN2*). As previously reported for fibroblasts^37^, we observed that at all developmental stages of the iPSC-MN differentiation essentially all *FL-SMN* transcripts from the sisters originated from their *SMN2* genes, even though the *SMN1* mutation is not expected to affect the transcription of the mutated gene (Supplementary Fig. S11). This points to possible nonsense–mediated decay of mutated *SMN1* transcripts in this family.

To perform statistical assessments of SMN-related parameters we compared the individuals of study by pooling together the values of their various cell types in the iPSC-MN differentiation process. Two parameters were highly consistent between cell types and showed strong albeit partial discriminatory power: *FL/Δ7-SMN* mRNA ratio and *tSMN* mRNA (Fig. 7C,D). The *FL/Δ7-SMN* mRNA ratio showed a statistically significant higher value in the 4603 control individual with two functional *SMN1* alleles compared to the carrier mother with one functional allele and the patients with no functional *SMN1* allele (p < 0.01- 0.001, Fig. 7C). *tSMN* mRNA showed a statistically significant reduction in type I SMA samples compared to those from the 4603 control and all family members (p <0.05- 0.001, Fig. 7D). For both parameters, samples from the carrier mother clustered with those from her type IIIa and type IV daughters rather than with the 4603 control, suggesting further effects of genotype.

**Figure 8:**
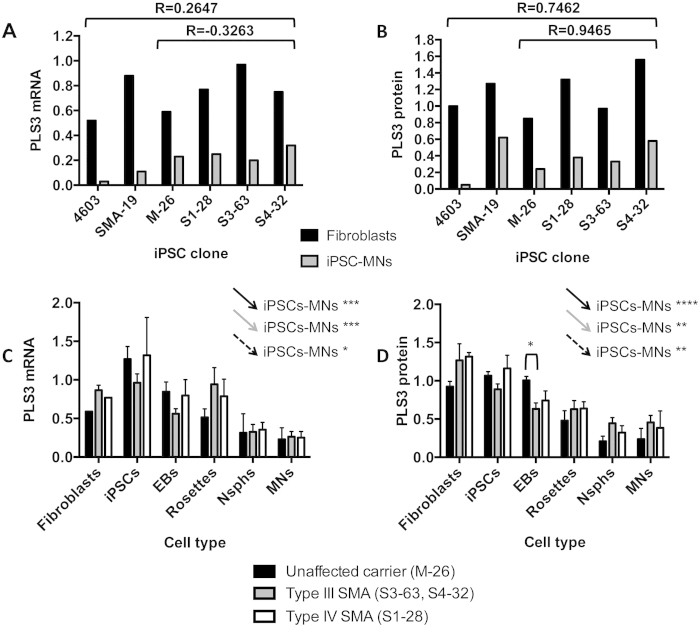
Comparative assessment of PLS3 measurements. (**A**) The expression of *PLS3* mRNA and (B) the level of protein in paired fibroblast and their corresponding differentiated MN cultures were compared in all individuals under study; *n* = 2. Parental fibroblasts from iPSC clone SMA-19 were not available and in this case fibroblasts from another type I SMA patient (with two *SMN2* copies and no *SMN1*) were used as an alternative reference. Pearson correlation coefficients (R) for values in fibroblasts and MNs are shown for all individuals combined and for the type IIIa/IV family. *PLS3* mRNA (**C**) and protein (**D**) values were compared between the female individuals of this study (carrier unaffected mother, type IIIa SMA siblings and type IV SMA sibling) across all cell types available. Arrows represent the statistical significance of the linear trend for gradual decrease along the MN differentiation process for each group. Data are presented as mean ± SEM. *p < 0.05; **p < 0.01; ***p < 0.001; ****p < 0.0001. Abbreviations: EBs, embryoid bodies; Nsphs, differentiating neuroepithelial-derived spheres; MNs, motor neurons.

We also compared *PLS3* mRNA and protein levels in fibroblasts from our subjects of study and their iPSC-derived MNs, again finding no correlation (Fig. 8A,B). Fibroblast values were higher than those in MNs for all individuals, both at mRNA and protein level. Given that PLS3 has been associated with a protective role in female SMA patients, we also performed a restricted comparison of *PLS3* mRNA and protein values in fibroblast and MN samples from the unaffected M-26 carrier mother, the SMA type III S3-63 and S4-34 and the type IV S1-28 daughters, excluding the male samples. No correlation was found (Fig. 8A,B). Extending the *PLS3* mRNA and protein level comparison to all cell types analysed and grouping the female samples as unaffected, type III or type IV SMA, we found no differences between groups, the exception being a statistically significant higher PLS3 protein level in the EBs of the unaffected carrier compared to the type III SMA siblings (Fig. 8C,D). In this all-female analysis we confirmed the significant linear trends for decreases in *PLS3* mRNA and protein levels along the iPSC to MN differentiation (Fig. 8C,D), first noted when all subjects were included (Fig. 6A,B). Furthermore, it does not seem likely that the type IV/asymptomatic phenotype of S1-28 is associated with high PLS3 levels, given that both transcript and protein levels are within the range observed in the other SMA samples (Fig. 8A,B).

## Discussion

The ability to differentiate iPSCs *in vitro* allows developmental studies as well as the establishment of disease models^27^,^28^,^29^,^30^. In our study, fibroblasts from two SMA type IIIa sisters, their haploidentical SMA type IV/asymptomatic sibling and their unaffected mother (carrier of the same *SMN1* mutation) were reprogrammed and differentiated into MNs. In this family, first described in 1995^26^, SMN protein expression^21^, *SMN2* copy number, *SMN2* transcript levels^37^ or *PLS3* levels^25^ do not explain the difference in phenotypes.

The iPSC lines produced from the affected family in the present work have been thoroughly characterised and subsequently differentiated towards the MN lineage using an established protocol^32^. The differentiating Nsphs thus produced showed the typical radial projection of TUJ1+ neurites upon attachment and were positive for MN markers ISL-1 and ChAT. Transduction with a lentiviral reporter driven by the MN-specific HB-9 promoter confirmed MN identity and the same temporal pattern as in differentiating hESCs^36^. For phenotypic analyses we used differentiating MN cultures, in which compact Nsphs contained cells expressing relevant MN markers. By D28 of differentiation most of the cells from all iPSC clones in this study were positive for the MN precursor marker OLIG2 (and p75-NGFR). Accurate quantification of MN yield was prevented by the EB-dependent MN differentiation method employed, but semi-quantitative estimates based on the presence of ISL-1 protein or HB9-driven expression suggested these to be present in ~10–15% of the population. We attempted to obtain isolated MNs^28^,^30^ but in our hands the viability of the single cells was severely affected and no downstream analyses were possible. It must be noted that a very recent 14-day protocol has described the differentiation of human MNs from human iPSCs cultured as single cells, with a yield of up to 74%^38^. However, the presence of a heterogeneous population in the MN cultures might be advantageous for the observation of SMA-related phenotypes. A recent study has shown, both in SMA mice *in vivo* and in human iPSC-derived SMA models, that SMA astrocytes show morphological and functional signs of activation preceding MN death^39^. These results are congruent with the need for global restoration of *SMN* expression to normalize survival in SMA mouse models^40^,^41^,^42^,^43^.

Three groups have previously reported the generation and MN differentiation of SMA type I iPSCs^27^,^28^,^29^,^30^. Although with different timings, these groups have observed disease phenotypes including lower proportion/number of MNs after differentiation^27^,^28^,^29^,^30^, decreased total MN cell body area^27^,^28^,^30^, delayed neurite outgrowth^29^, decreased number of neurites^28^, shorter MN axons and growth cones^30^ and presynaptic defects^27^. We investigated MN cultures differentiated from representative chronic SMA iPSCs. The analysis of normalised neurite number and neurite length during the early period of MN differentiation (D27–D34) suggested that the SMA type IV/asymptomatic clone S1-28 resembled the maternal carrier clone M-26 more closely than the more affected type IIIa clones S3-63 and S4-32. These results suggest that the pathology and severity observed in the *in vitro* models of SMA may recapitulate developmental events *in vivo* in affected individuals^44^.

Research in SMA mouse models (reviewed in^12^) and in human samples^44^, suggests that the synaptic organization of NMJs is affected in SMA. Co-culture systems of myotubes and MNs are attractive as a way to dissect the structure and function of the NMJ^45^, and in such cultures type I SMA iPSC–derived MNs have been reported to produce fewer and smaller endplates compared to heterozygote controls^30^. We prepared similar cultures and observed a clearly different pattern in aneural cultures (differentiating myoblasts with no MN preparations added) compared to co-cultures including differentiating MNs from the type IIIa/IV family. However, no differences were observed among the co-cultures of clones from the affected type IIIa/IV family, which may be due to a genuine lack of *in vitro* NMJ phenotype in these milder forms of the disease or to the relatively short time-frame in which the co-cultures were assessed.

SMA is the result of a decrease in the amount of SMN protein, but the increased susceptibility of MNs is not understood. Developmental reductions in levels of SMN in human brain and animal spinal cord have been reported^46^,^47^,^48^,^49^,^50^, although some examples of apparently steady levels in spinal cord and specifically in MNs have also been published^50^,^51^,^52^. Several lines of study in SMA models have suggested that SMA is a developmental disorder, at least in the most severe cases^53^,^54^,^55^,^56^,^57^,^58^, and studies on human foetuses have provided significant evidence of prenatal onset of pathological defects in predicted type I SMA disease^44^,^59^,^60^,^61^. We followed the levels of SMN protein during the *in vitro* differentiation of iPSCs to MNs and found a statistically significant reduction, independent of sex, disease state or severity. This observation recapitulates the developmental reduction in spinal cord SMN detected in most models and suggests that MNs may complete differentiation with about 2-fold reduced levels of the protein. Whether the possible developmental reduction in SMN predisposes MNs to the selective defects and sensitivity observed in SMA remains to be demonstrated but it is an interesting working hypothesis.

Transcriptional analyses of *SMN* genes show less consistency. Down-regulation of *SMN* promoter activity has been observed on *in vitro* differentiation of embryonic carcinoma cells towards the neuronal lineage^62^, but increases in *FL-SMN* and *Δ7-SMN* transcripts have been described during differentiation of an hESC clone towards MNs following the same protocol employed here^63^. Our own analyses using a variety of iPSC clones and differentiation batches show significant variability in *FL-SMN* and *Δ7-SMN* transcripts, in most cases showing a bell-shaped distribution, while *tSMN* transcripts remain fairly constant (Supplementary Fig. S9). Of note, wild-type iPSC clone 4603 showed increased levels of *FL-SMN* transcript during differentiation, similar to the reported wild-type hESC clone^63^, so it is possible that disease status has a bearing. However, as previously described^37^,^64^, we found no correlation between *FL-SMN* transcript and protein levels in our iPSC differentiation studies, and protein levels were not reported in the hESC study^63^.

*PLS3* has been reported to have a modifier role for SMA in discordant female siblings^22^,^23^,^24^,^65^,^66^, perhaps due to its involvement in actin dynamics at the NMJ^22^,^65^. We have previously found no correlation between SMA severity and *PLS3* expression in whole blood, lymphoblasts or fibroblasts of the family studied here^25^. We have now compared transcript and protein levels across the various stages of iPSC-MN differentiation, observing a statistically significant reduction in the levels of both mRNA (5-fold) and protein (2-fold) along the differentiation process, irrespective of sex, disease state or severity. These findings contrast with the previously reported increase in *PLS3* expression found during the neural differentiation of PC12 cells^22^ and further support the lack of a modifier role for *PLS3* in the SMA family under study.

One of the main aims for SMA therapeutic research is the definition of reliable biomarkers, ideally measured in surrogate samples or cells of easy access^64^,^67^,^68^,^69^,^70^,^71^,^72^. *SMN2* copy number has consistently shown high correlation with SMA type^13^,^14^, except in discordant SMA families such as the one studied here^15^,^16^,^17^,^18^,^19^,^20^,^21^,^73^. Measurements of *SMN* transcripts and protein do not clearly distinguish between the diversity of SMA phenotypes^10^,^64^,^67^,^68^,^69^,^70^,^71^, as we have confirmed here in iPSC-derived MNs. Using our dataset from iPSC-MN differentiation studies we concur that the *FL/Δ7-SMN* mRNA ratio is a robust parameter to distinguish control individuals with two functional *SMN1* alleles and SMA patients, as described in cell cultures, foetal tissues and adult whole blood samples^10^,^37^,^64^, but our results also indicate that it is not suitable to differentiate wild-type from the carrier mother. We have also confirmed that *tSMN*, a parameter that takes into consideration all isoforms of *SMN* transcript, distinguishes the type I SMA individual from all other subjects, as previously reported for peripheral blood leucocytes^64^,^69^. This is consistent with the minimal number of functional *SMN* gene copies in severe SMA. Finally, our analyses of *PLS3* transcript and protein levels show no usefulness for these parameters as SMA biomarkers.

In summary, we have produced iPSCs from a discordant type IIIa/IV SMA family and differentiated them to MN populations, observing more modest neurite defects than previously reported for severe type I SMA. SMN protein analyses during the iPSC-MN differentiation process revealed a progressive decrease, which recapitulates previous developmental observations. *PLS3* underwent similar reductions at transcriptional and translational levels. SMN and PLS3 reductions may be implicated in the increased vulnerability of MNs in SMA disease, but PLS3 levels in iPSC-derived MNs do not explain the phenotypic discrepancy between siblings of the discordant SMA family under study. Our measurements of *SMN* transcripts in iPSC-derived MN populations have confirmed their limited value as SMA biomarkers.

## Materials and methods

### Patient samples

We studied five individuals from a consanguineous Spanish SMA family (Fig. 1A and Supplementary Table S2). The unaffected mother (M), is a heterozygous carrier of the *SMN1* exon 3 mutation (c.399_402delAGAG). Patients S2, S3 and S4 suffer from proximal SMA type IIIa as defined by the criteria of the International SMA consortium^5^,^74^. S1 was virtually asymptomatic until her thirties and at present walks normally, having minimum clinical and electromyographic manifestations. All sisters have four *SMN2* copies^15^. Fibroblasts were obtained from fresh skin biopsies for all individuals except for S2, now deceased, from whom a frozen fibroblast sample was used but failed to generate iPSC clones. Biopsies were taken with the understanding and written consent of each subject, using methods carried out in accordance with the approved guidelines and following experimental protocols approved by the Hospital de la Santa Creu i Sant Pau Clinical Research Ethics Committee. Clone 4603 (wild-type) and SMA-19 (type I SMA,^29^) are male iPSCs (Supplementary Table S2).

### Cell culture, vector production, fibroblast reprogramming and iPSC differentiation

#### Cell line culture

Primary human fibroblasts were maintained in Dulbecco’s modified Eagle’s medium high glucose with stable glutamine (DMEMg), supplemented with 10% foetal bovine serum (FBS), 1 mM sodium pyruvate, 1X non-essential amino acids (NEAAs), 1 mM L-Ascorbic acid 2-phosphate (AA2P) and 10 ng/ml fibroblast growth factor 2 (FGF_2_). PLAT-E cells were maintained in DMEMg, supplemented with 10% FBS, 1 mM sodium pyruvate and 50 μM β-mercaptoethanol. HEK293T cells and C2C12 mouse myoblasts (ATCC, CRL-1772) were maintained in DMEMg, supplemented with 10% FBS. To induce the formation of C2C12 myotubes, FBS was replaced by 2% horse serum, and cells were cultivated in confluence. iPSC clones were maintained on mytomycin C growth-arrested immortalized human BJ1 fibroblasts (Clontech, Cat. No. C4001-1) in Stemedia NutriStem XF/FF Culture Medium (Stemgent, Cat. No. 130-095-543).

#### Vector production

Recombinant Moloney Murine Leukemia viral vectors (rMMLVs) were produced by the independent transfection of *OCT4, SOX2, KFL4 AND c-MYC* plasmids (Addgene plasmids 17220, 17225, 17226 and 17227) into PLAT-E packaging cells (Cell Biolabs). HIV-1 based lentiviral vectors (rH1LVs) were produced by co-transfection of pMDLg/pRRE, pRSV-REV, pMD2.VSV-G and pCCLsc_HB9_RFP_W in HEK293T cells as previously described^75^. pCCLsc_HB9_RFP_W was kindly provided by Prof Fred Gage (Salk Institute, USA).

#### iPSC reprogramming and culture

Fibroblasts were transduced with a 1:1:1:1 mix of the four rMMLVs after incubation with murine cationic amino acid transporter-1 (mCAT-1) gesicles as previously described^76^. Valproic acid (VPA) supplementation was used for the first ten days. Potential iPSC colonies were selected on the basis of their embryonic stem cell (ESC) like-morphology. Clones that survived and presented robust growth were expanded and frozen.

#### Embryoid body (EB) formation

iPSCs were harvested by dispase treatment and grown in suspension for seven days. EBs were further plated in 0.1% gelatin-coated 24 well plates and cultured for seven more days before fixation.

#### MN differentiation and transduction

MN differentiation was performed following a well–established, EB-based protocol originally developed for hESCs^32^. In brief, iPSCs were induced to neuroepithelial cells in the absence of morphogens in the first 2 weeks by generating EBs in the first week and plating them in neural differentiation medium (DMEM Nutrient mix F12 supplemented with 1X N2, 1X NEAAs and 2 μg/ml heparin) in the second week. Between days 10–17 neural tube like-rosettes became apparent within the plated clusters, and they were lifted by mechanical selection and grown in suspension as differentiating Nsphs for two weeks in the presence of 0.1 μM retinoic acid (RA) and 1 μM purmorphamine. The aggregates were split by incubation with accutase when their diameter increased above 300 μm. Differentiating Nsphs containing MN progenitors were plated at day 28 on Poly-L Ornithine-Laminin (PO-Lam) coated surfaces and cultured in neural differentiation medium supplemented with 50 nM RA, 0.5 μM purmorphamine, 0.1 μM cyclic adenosine monophosphate (cAMP), 200 ng/ml AA2P, 10 ng/ml brain-derived neurotrophic factor (BDNF), 10 ng/ml glial cell-derived neurotrophic factor (GDNF) and 10 ng/ml insulin-like growth factor 1 (IGF-1) to generate post-mitotic MNs. This medium is referred to as complete MN differentiation medium and it was changed every other day. For transduction with rH1LVs, four D22 Nsphs were incubated in a microfuge tube for 2 hours (37 °C) in 200 μl of neural differentiation medium containing lentivector at a qPCR MOI of 200 before plating.

#### Co-culture system

iPSC-derived MN preparations were cultured on mice myotubes in order to observe the formation of NMJ-like structures. For this, C2C12 myoblasts were induced to differentiate in laminin-coated permanox TC chamber slides for four days. Four to six differentiating D39 Nsphs were then seeded on myotubes in a 1:1 mix of C2C12 differentiation medium and complete MN differentiation medium. Cells were then cultured for 3 days before fixation and staining with α–bungarotoxin (for AChRs) and TUJ1 antibody (for neurites). The average size of AChR clusters was measured on captured images by manually demarcating the regions of interest based on the homogeneity and distribution of the AChR signal. Measurements were performed in at least five different fields from the iPSC-derived MNs/C2C12 myoblast co-cultures of each clone; fields were selected in areas where neurites were present. Control measurements were also performed in fields where there was no direct contact between myotubes and the Nsphs or the neurites extending from them as well as in aneural cultures.

### Neurite length measurement

Brightfield images of 5 small Nsphs (150–200 μm in diameter) per clone were taken on D27, D28, D29, D32 and D34 with an inverted Axio observer.A1 microscope (Zeiss) using a 2X objective. The number and length of all neurites and the surface area of the Nsphs were measured by a blinded observer using ImageJ software (NIH, MD, USA). The number of neurites per Nsph was normalised to the area of the corresponding Nsph. The values of normalised neurite numbers and neurite length were averaged for each Nsph separately. Neurites in large bundles were counted if they could be individually distinguished. Neurite length was measured from the edge of the cluster to the visually distinguishable end point. Neurite growth within the cluster was not assessed.

### Immunocytochemistry

Cells were fixed and stained with primary and secondary antibodies (Supplementary Table S3) following standard procedures. Images were taken with an inverted fluorescent Zeiss Axio observer.A1 microscope or an SP5 Leica confocal microscope. To estimate the yield of MNs in differentiated Nsphs preparations we counted total nuclei (using DAPI) and ISL-1 positive nuclei in areas where this was feasible, using imageJ (NIH) for both automatic processing and manual quantification.

### Alkaline Phosphatase (AP) staining

Cells were fixed, stained with SIGMAFAST™ (Sigma-Aldrich. Cat No. B5655), washed and dried following manufacturer’s recommendations. Images were taken with a Leica DM IRB microscope.

### RNA isolation, cDNA synthesis, *SMN* transcripts origin and RNA expression analysis

#### RNA isolation

RNA was extracted with TRIZOL (Invitrogen) following manufacturer’s instructions.

#### cDNA synthesis

1 μg of the corresponding RNA, and Moloney Murine Leukemia Virus Reverse Transcriptase (MMLV, Promega) were used.

#### Origin of SMN transcripts

A segment corresponding to exons 6–8 was amplified by PCR, followed by *Dde*I restriction as previously described^10^.

#### RNA expression analysis and normalisation method

Reverse transcription polymerase chain reaction (RT-PCR) and quantitative RT-PCR (qRT-PCR) analyses for the assessment of down regulation of transgenes in iPSCs were performed using previously described primers^77^. RT-PCRs were performed in a PCR gradient Thermal cycler TC-512 (Techne) using GoTaq Flexi DNA Polymerase (Promega) and qPCRs were performed in a Rotor-Gene 6000 real time rotary analyser (Corbett Life Science) using SensiMixPlus SYBR (Quantance). *SMN* and *PLS3* transcripts were quantified by qRT-PCR employing custom-made and commercial TaqMan probes^37^. Samples were amplified and analysed in an ABI PRISM® 7900HT Sequence Detection System. A cDNA sample of SMA type II fibroblasts was systematically included along with the samples of study during the amplification of each target gene and its value arbitrarily set to one in order to be used as calibrator. The stability of at least six different housekeeping genes was analysed across all cell types included in the analyses using the geNorm VBA applet for Microsoft Excel^78^. All qRT-PCR values were normalised to the geometric mean of the most stable reference genes determined across all pertinent samples^78^: *ribosomal protein L13a (RPL13A)* and *ribosomal RNA 18S (18S)* for the assessment of transgene expression in iPSCs, and *beta actin (ACTB), peptidylprolyl isomerase A (PPIA)* and *glyceraldehyde-3-phosphate dehydrogenase (GAPDH)* for the quantification of *SMN* and *PLS3*. All primers were validated (i) by *in silico* specificity, (ii) by empirical specificity, through the amplification of a single product of the expected size, as observed in agarose gels; and (iii) in a qRT-PCR/qPCR run of serial dilutions of a control sample showing amplification efficiencies of 100% ± 10%, slopes of 3.3 ± 10% and slopes CT target gene/CT reference gene of less than 0.1. A list of all primers used is shown in Supplementary Table S4.

### Western Blot

Samples were harvested and Western blotting performed using standard procedures, with the antibodies listed on Supplementary Table S3. Detection and quantification of proteins was performed with an Odyssey Infrared Imaging System and application software V1 (LI-COR Biosciences). Target protein levels were normalised to the geometric mean of the values from alpha tubulin (TUBA), glucose phosphate isomerase (GPI1) and actin. A protein sample from SMA type II fibroblasts was systematically included along the samples of study and its value arbitrarily set to one in order to be used as calibrator.

### Flow cytometry

Cells co-expressing surface makers SSEA3 and TRA-1-81 were quantified by flow cytometry in a BD FACSCanto II with the BD FACSDiva Software (BD Biosciences).

### Karyotyping

G-banding and mFISH were performed on metaphase chromosomes according to standard protocols.

### DNA fingerprinting

Cell identity was verified by comparing the short tandem repeat (STR) profile at several loci. The profile was compared between iPSC clones, the original fibroblasts used for reprogramming and peripheral blood samples from the patients. The STRs studied were: D1S305 (chromosome 1), D2S443 and D2S291 (chromosome 2), D19S112 and D19S562 (chromosome 19) and INT25-2.0 (chromosome X).

### Teratoma formation

The teratoma assay was performed by Applied Stem Cell, Inc. (ASC, California, USA) following standard procedures. Methods were carried out in accordance with the approved guidelines, and all experimental protocols were approved by ASC’s Institutional Animal Care and Use Committee (IACUC).

### Statistical analyses

GraphPad Prism software was used for statistical analyses. Data were presented as mean ± standard error of the mean (SEM). Two-way ANOVA with Bonferroni’s *post hoc* test was used to analyse normalised neurite number, neurite length, *SMN* and *PLS3* expression. For the analyses of linear trends of *SMN* and *PLS3* expression one-way ANOVA with Dunnet’s and linear trend *post hoc* tests were employed. Kruskal-Wallis with Dunn’s *post hoc* test was used for the analysis of the area of AChR clusters. For correlation analyses Pearson correlation coefficients were calculated. Differences were considered statistically significant when the P value was <0.05 (*), <0.01 (**), <0.001 (***) or <0.0001 (****).

## Additional Information

**How to cite this article**: Boza-Morán, M. G. *et al.* Decay in survival motor neuron and plastin 3 levels during differentiation of iPSC-derived human motor neurons. *Sci. Rep.* **5**, 11696; doi: 10.1038/srep11696 (2015).

## Supplementary Material

Supplementary Information
