# Supplementary material for: Decay in survival motor neuron and plastin 3 levels during differentiation of iPSC-derived human motor neurons
Source: Sci Rep. 2015 Jun 26;5:11696. doi: 10.1038/srep11696 (PMC4650562; doi:10.1038/srep11696)
Supplement: Supplementary Information [file srep11696-s1.pdf]

Decay in survival motor neuron and plastin 3 levels during differentiation of iPSC-derived human motor neurons

María G Boza-Morán[1], Rebeca Martínez-Hernández[1,2,*], Sara Bernal[2,*], Klaus Wanisch[1,*], Eva Also-Rallo[2], Anita Le Heron[1], Laura Alías[2], Cécile Denis[3], Mathilde Girard[3], Jiing-Kuan Yee[4], Eduardo F. Tizzano[2,‡,†] & Rafael J Yáñez-Muñoz[1,‡]

[1]School of Biological Sciences, Royal Holloway, University of London, Egham, TW20 0EX, UK. [2]Department of Genetics and CIBERER U-705, Hospital de la Santa Creu i Sant Pau, Barcelona, Spain. [3]Institute for Stem Cell Therapy and Exploration of Monogenic Diseases, Evry Cedex, France. [4]Department of Virology, Beckman Research Institute, City of Hope, Duarte, California, USA. [*]These authors contributed equally to this study. [‡]E.F.T. and R.J.Y.-M. are the senior authors in this study. [†]Present address: Department of Clinical and Molecular Genetics, Hospital de Vall d'Hebron, Barcelona, Spain. Correspondence and requests for materials should be addressed to R.J.Y.-M. (email: rafael.yanez@rhul.ac.uk)

SUPPLEMENTARY INFORMATION

Supplementary Table S1. STR profile of selected iPSC clones, their parental fibroblasts and peripheral blood from the donors. Values shown correspond to number of repeats.

	Chrom 1 D1S305	Chrom 2 D2S443	Chrom 2 D2S291	Chrom 19 D19S112	Chrom 19 D19S562	Chrom X INT25-2.0
**M-26 iPSCs**	25, 27	11, 13	18, 18	11,12	13, 33	15, 17
**M Fibroblasts**	25, 27	11, 13	18, 18	11,12	13, 33	15, 17
**M Peripheral blood**	25, 27	11, 13	18,18	11,12	13, 33	15, 17
**S1-28 iPSCs**	27, 27	13, 13	18, 26	11,12	13, 33	15, 16
**S1 Fibroblasts**	27, 27	13, 13	18, 26	11,12	13, 33	15, 16
**S1 Peripheral blood**	27, 27	13, 13	18, 26	11,12	13, 33	15, 16
**S3-63 iPSCs**	27, 31	13, 13	18, 26	11, 16	13, 27	16, 17
**S3 Fibroblasts**	27, 31	13, 13	18, 26	11, 16	13, 27	16, 17
**S3 Peripheral blood**	27, 31	13, 13	18, 26	11, 16	13, 27	16, 17
**S4-32 iPSCs**	25, 31	11, 12	18, 18	12, 12	33, 33	15, 16
**S4 Fibroblasts**	25, 31	11, 12	18, 18	12, 12	33, 33	15, 16
**S4 Peripheral blood**	25, 31	11, 12	18, 18	12, 12	33, 33	15, 16

Supplementary Table S2. Clinical, molecular and cellular data of the individuals included in this study and the iPSCs derived from them. NA: not applicable. ND: not determined. Data concerning the members of the Spanish family of study (M, S1, S3 and S4) have been previously described (Bernal *et al.*, 2011; Also-Rallo *et al.*, 2011).

**Individual**	**4603**	**SMA-19**	**M**	**S1**	**S3**	**S4**
**Race**	Caucasian	Caucasian	Caucasian	Caucasian	Caucasian	Caucasian
**Sex**	Male	Male	Female	Female	Female	Female
**Genotype**	NA	Homozygous for deletion of exons 7 and 8 of the *SMN1* gene	Carrier of c.399_402delAGAG mutation in *SMN1* gene	Homozygous for c.399_402delAGAG mutation in *SMN1* gene	Homozygous for c.399_402delAGAG mutation in *SMN1* gene	Homozygous for c.399_402delAGAG mutation in *SMN1* gene
***SMN2* copy number**	ND	2	3	4	4	4
**Origin of *FL-SMN* transcripts**	*SMN1* and *SMN2*	All from *SMN2*	*SMN1* and *SMN2*	Mostly from *SMN2*	Mostly from *SMN2*	Mostly from *SMN2*
**Age of onset of weakness**	NA	<6 months	NA	NA	18 months	18 months
**Wheelchair bound (age they stopped being able to walk)**	NA	Never walked	NA	NA	14 years	8 years
**Electromyography results**	NA	Denervation	NA	Minimal changes	Denervation	Denervation
**Diagnosis**	Unaffected	SMA type I	Unaffected	Asymptomatic or SMA type IV	SMA type IIIa	SMA type IIIa
**Cell type used for iPSC reprogramming**	Fibroblasts harvested from skin biopsy. Coriell Cat No GM04603	Fibroblasts harvested from eye lens. Coriell Cat No GM09677	Fibroblasts harvested from skin biopsy	Fibroblasts harvested from skin biopsy	Fibroblasts harvested from skin biopsy	Fibroblasts harvested from skin biopsy
**Age at sample collection**	Adult. Not Specified	2 years	>60	45	38	36
**iPSC clone selected**	4603. Unpublished, iSTEM (France)	SMA-19. *Chang et al., 2011*	M-26	S1-28	S3-63	S4-32

Supplementary Table S3. Antibodies used in the present study.

Antibody name	Manufacturer	Catalogue number	Concentration/Dilution	Technique
Alexa Fluor® 647 anti-human SSEA-3	BioLegend	330308	0.125 µg/ $5x10^5$ cells	Flow cytometry
Alexa Fluor® 647 Rat IgM, κ Isotype Ctrl	BioLegend	400813	0.125 µg/ $5x10^5$ cells	Flow cytometry
PE anti-human TRA-1-81	BioLegend	330708	0.125 µg/ $5x10^5$ cells	Flow cytometry
PE Mouse IgM, κ Isotype Ctrl	BioLegend	401609	0.125 µg/ $5x10^5$ cells	Flow cytometry
Mouse anti-OCT4	Chemicon	MAB4401	1 in 500	Immunocytochemistry
Mouse anti-TRA-1-60	Chemicon	MAB4360	1 in 500	Immunocytochemistry
Rabbit anti-SOX2	Chemicon	AB5603	1 in 500	Immunocytochemistry
Mouse anti NANOG	Abcam	AB62734	1 in 100	Immunocytochemistry
Mouse anti-Smooth muscle actin (SMA)	DAKO	M0851	1 in 100	Immunocytochemistry
Rabbit anti-Desmin	Abcam	AB15200	1 in 200	Immunocytochemistry
Rabbit anti-Alpha Fetoprotein	Molecular Probes	18-0055	1 in 100	Immunocytochemistry
Goat anti-SOX17	R&D systems	AF1924	1 in 100	Immunocytochemistry
Goat anti-SOX1	R&D systems	AF3369	1 in 500	Immunocytochemistry
Mouse anti-PAX6	DSHB	Supernatant	1 in 500	Immunocytochemistry
Goat anti- ISLET1	R&D systems	AF1837	1 in 50	Immunocytochemistry
Goat anti-Choline acetyltransferase (ChAT)	Chemicon	AB144P	1 in 50	Immunocytochemistry

Rabbit Anti-Neuronal class III beta tubulin (TUJ1)	Sigma	T 2200	1 in 3000	Immunocytochemistry
α-Bungarotoxin, Alexa Fluor® 555 Conjugate	Molecular Probes	B35451	1 in 500	Immunocytochemistry
Alexa Fluor 568 donkey anti -mouse IgG	Molecular probes	A10037	1 in 1000	Immunocytochemistry
Alexa Fluor® 488 donkey anti-mouse IgG (H+L)	Molecular probes	A21202	1 in 1000	Immunocytochemistry
Alexa Fluor® 488 donkey anti-goat IgG (H+L)	Molecular probes	A-11055	1 in 1000	Immunocytochemistry
Alexa Fluor® 546 donkey anti-goat IgG (H+L)	Molecular probes	A11056	1 in 1000	Immunocytochemistry
Alexa Fluor® 555 goat anti-rabbit IgG (H+L)	Molecular probes	A21428	1 in 1000	Immunocytochemistry
Alexa Fluor® 488 goat anti-rabbit IgG (H+L)	Molecular probes	A-11034	1 in 1000	Immunocytochemistry
Mouse anti-survival motor neuron (SMN)	BD Biosciences	610646	1 in 5000	Western blot
Rabbit Anti-T Plastin antibody	ABCAM	AB128690	1 in 1000	Western blot
Rabbit anti-Alpha tubulin	ABCAM	AB4074	1 in 2000	Western blot
Mouse anti-Actin	DSHB	JLA20. Supernatant	1 in 100	Western blot
Mouse anti- Glucose phosphate isomerase (GPI1)	DSHB	CPTC-GPI-1. Supernatant	1 in 100	Western blot
IRDye 800CW goat anti-mouse	LI-COR	92632210	1 in 2000	Western blot
Goat anti-Rabbit alexa flour 680	Molecular Probes	A21076	1 in 5000	Western blot

Supplementary Table S4. Primer sequences for RT-PCR/qPCR reactions.

Gene	Forward primer sequence (5’ → 3’)	Reverse primer sequence (5’ → 3’)	Taqman Probe (5’ → 3’)	Reference
c-MYC endogenous	TGCCTCAAATTGGACTTTGG	GATTGAAATTCTGTGTAACTGC		Park *et al.*, 2008
c-MYC total	ACTCTGAGGAGGAACAAGAA	TGGAGACGTGGCACCTCTT		Park *et al.*, 2008
c-MYC transgene	TGCCTCAAATTGGACTTTGG	CGCTCGAGGTTAACGAATT		Park *et al.*, 2008
OCT4 endogenous	CCTCACTTCACTGCACTGTA	CAGGTTTTCTTTCCCTAGCT		Park *et al.*, 2008
OCT4 total	AGCGAACCAGTATCGAGAAC	TTACAGAACCACACTCGGAC		Park *et al.*, 2008
OCT4 transgene	CCTCACTTCACTGCACTGTA	CCTTGAGGTACCAGAGATCT		Park *et al.*, 2008
KFL4 endogenous	GATGAACTGACCAGGCACTA	GTGGGTCATATCCACTGTCT		Park *et al.*, 2008
KFL4 total	TCTCAAGGCACACCTGCGAA	TAGTGCCTGGTCAGTTCATC		Park *et al.*, 2008
KFL4 transgene	GATGAACTGACCAGGCACTA	CCTTGAGGTACCAGAGATCT		Park *et al.*, 2008
SOX2 endogenous	CCCAGCAGACTTCACATGT	CCTCCCATTTCCCTCGTTTT		Park *et al.*, 2008
SOX2 total	AGCTACAGCATGATGCAGGA	GGTCATGGAGTTGTACTGCA		Park *et al.*, 2008
SOX2 transgene	CCCAGCAGACTTCACATGT	CCTTGAGGTACCAGAGATCT		Park *et al.*, 2008
RPL13A	CTACGACAAGAAAAAGCGGA	GCCCCAGATAGGCAAAC		Designed here
18S	GAGGATGAGGTGGAACGTGT	TCTTCAGTCGCTCCAGGTCT		Designed here

SMN 6-8	CTCCCATATGTCCAGATTCTCTTG	CTACAACACCCTTCTCACAG		Soler-Botija *et al.*, 2005
Full-length Survival motor neuron (FL-SMN)	GCT GAT GCT TTGGGAAGTATGTTA	CACCTTCCTTCTTTTTGATTTTGTC	FAM-TTTCATGGTACATGAGTGGCTATCATACTGGCTATTAT-MGBNFQ	Also-Rallo *et al.*, 2011
SMN Delta 7 isoform (Δ7-SMN)	TGGACCACCAATAATTCCCC	ATGCCAGCATTTCCATATAATAGCC	FAM-ACCACCTCCCATATGTCCAGATTCTCTTGATG-MGBNFQ	Also-Rallo *et al.*, 2011
Total SMN (FL-SMN + Δ7-SMN)	NA			Applied Biosystems Cat No. Hs00165806_m1
Plastin 3	NA			Applied Biosystems Cat No. Hs00192406_m
Beta actin	NA			Applied Biosystems Cat Hs99999903_m1
Peptidylprolyl isomerase A	NA			Applied Biosystems Hs99999904_m1
Glyceraldehyde-3-phosphate dehydrogenase	NA			Applied Biosystems Cat No Hs02758991_g1

Supplementary Figure S1

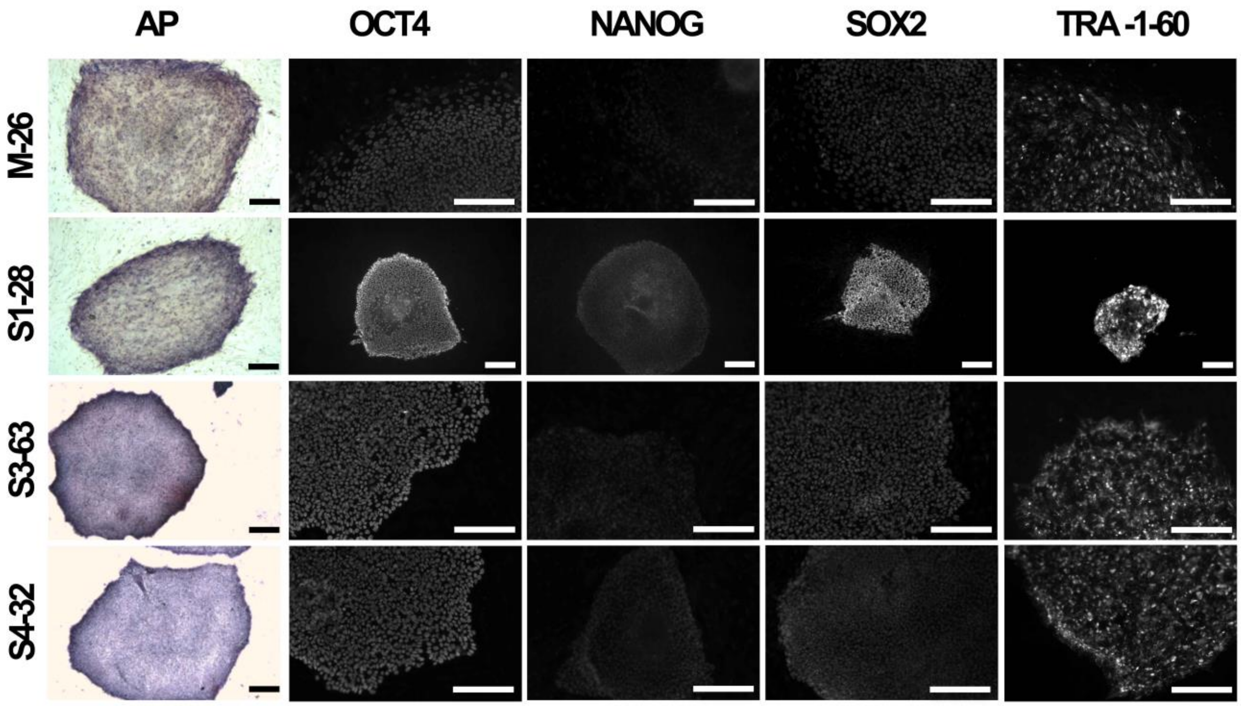

Supplementary Fig. S1. Expression of pluripotency markers in selected iPSC clones. Alkaline phosphatase (AP) staining and immunofluorescence analysis show the expression of the pluripotency markers AP, *OCT4, NANOG, SOX2,* and *TRA-1-60* in representative colonies of iPSC clones. Scale bar= 200 µm.

Supplementary Figure S2

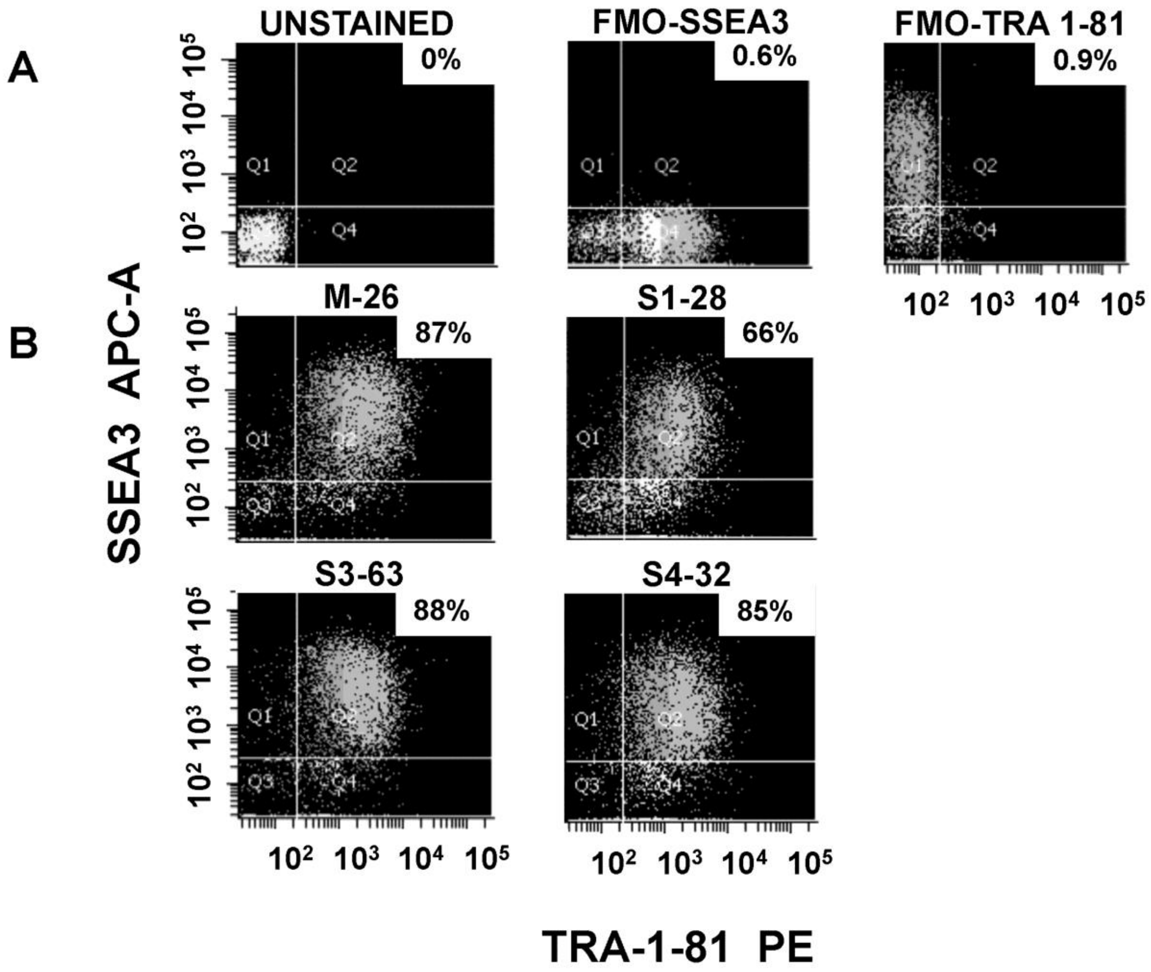

Supplementary Fig. S2. Quantitative assessment of pluripotency. Multi-parameter flow cytometry was performed on selected iPCS clones in order to determine the proportion of cells co-expressing the pluripotency markers SSEA3 and TRA 1-81. (A) Control samples after calibration and set up of appropriate thresholds. (B) The high percentage of double positive SSEA3/TRA1-81 cells in clones M-26, S3-63 and S4-32, indicated on the top right corner of the corresponding dot-plot, suggests low tendency for spontaneous differentiation in culture. Clone S1-28 shows a moderate propensity to differentiate. Abbreviations: FMO: fluorescence minus one.

Supplementary Figure S3

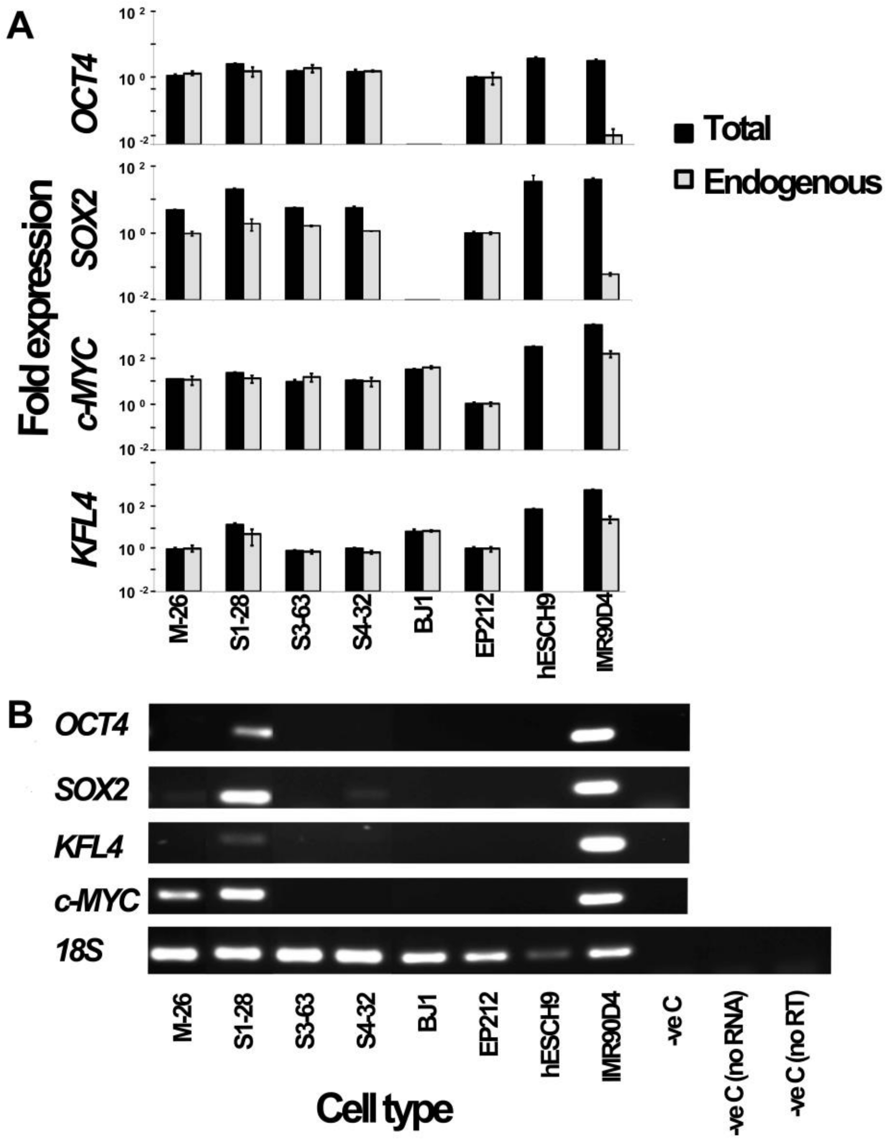

Supplementary Fig. S3. Down-regulation of transgenes in selected iPSCs. The down-regulation of the four transgenes, *OCT4*, *SOX2*, *c-MYC* and *KFL4*, was assessed by quantitative and standard reverse transcription (RT)-PCR. (A) The endogenous and total (endogenous + exogenous) expression of the pluripotency genes was quantified by qRT-PCR. Gene expression was normalized to the geometric mean of *RPL13* and *18S* and plotted (log10 scale) relative to the expression of EP2102 carcinoma cells, which was arbitrarily set to 1. Data are presented as mean ± standard deviation. (B) The expression of the transgenes was measured through RT-PCR with primers targeting retroviral-specific sequences. *18S* is shown as a positive amplification and loading control. BJ1 human fibroblasts (BJ1), IMR90 human fibroblasts undergoing reprogramming and harvested 4 days post transduction (IMR90 D4) and H9 human embryonic stem cells (hESCs) were included in the analysis as references.

Supplementary Figure S4

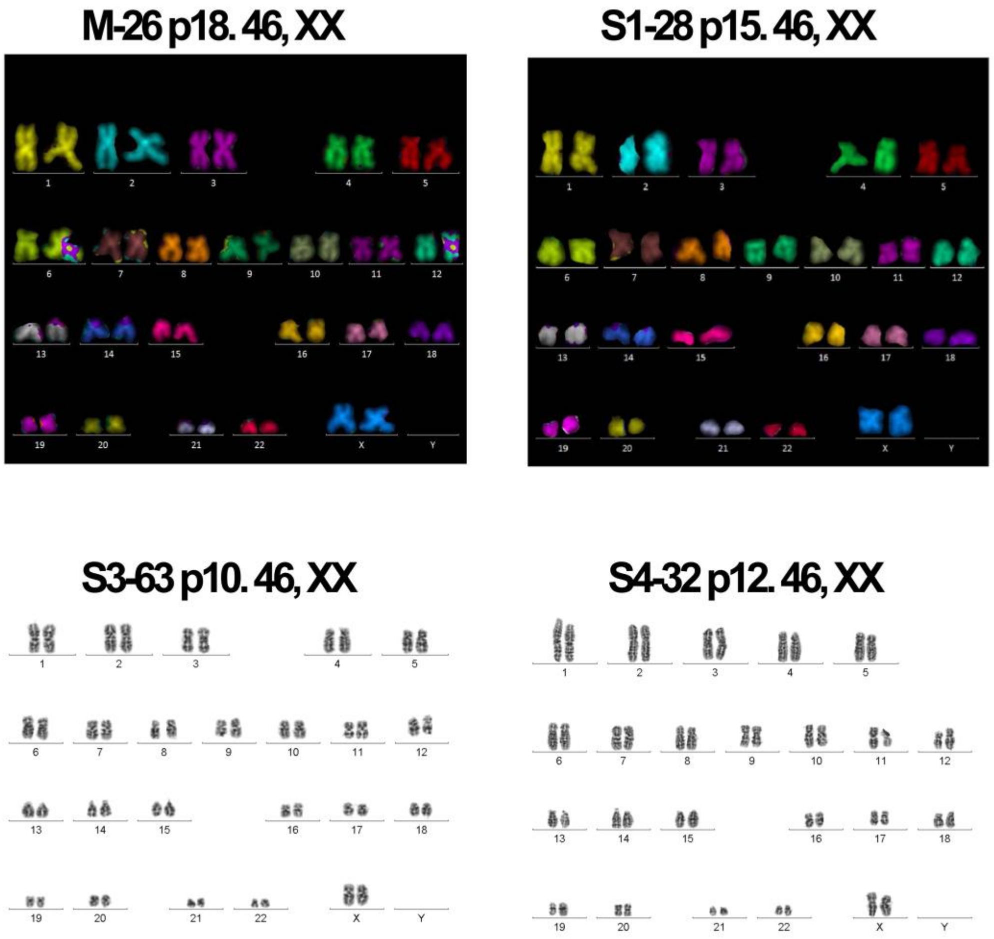

Supplementary Fig. S4. Karyotype analysis of selected iPSC clones. mFISH (clones M-26 and S1-28) and G-banding (clones S3-63 and S4-32) were performed on metaphase spreads. Clone name, passage number at the time of cell cycle arrest and cell fixation and karyotype are indicated.

Supplementary Figure S5

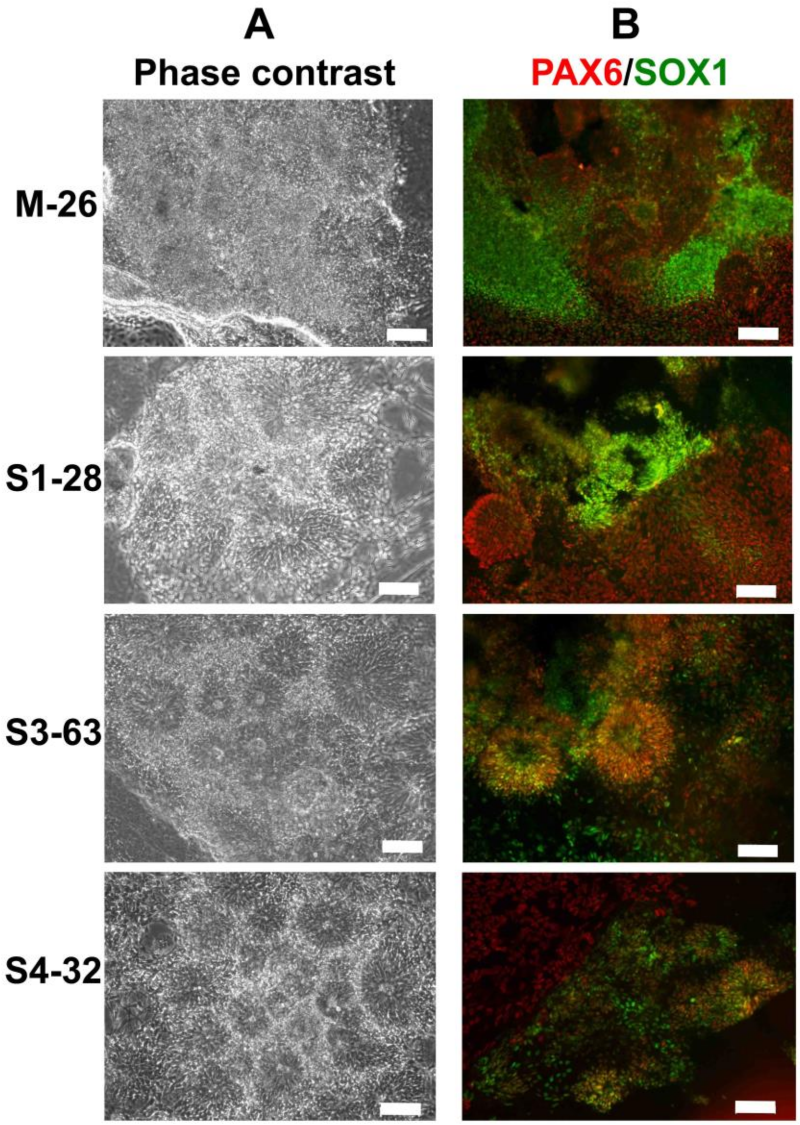

Supplementary Fig. S5. Rosette formation and neuroepithelial identity. Selected iPSC clones were grown as embryoid bodies (EBs) for seven days and then plated in gelatine-coated plates until rosette formation. (A) Several neural–tube like rosettes can be seen within each EB cluster 14-15 days after the start of the differentiation protocol. (B) Co-expression of PAX6 (red) and SOX1 (green) confirmed neuroepithelial identity. Images A and B for each clone were taken from different fields/wells. Scale bars = 100 μm.

Supplementary Figure S6

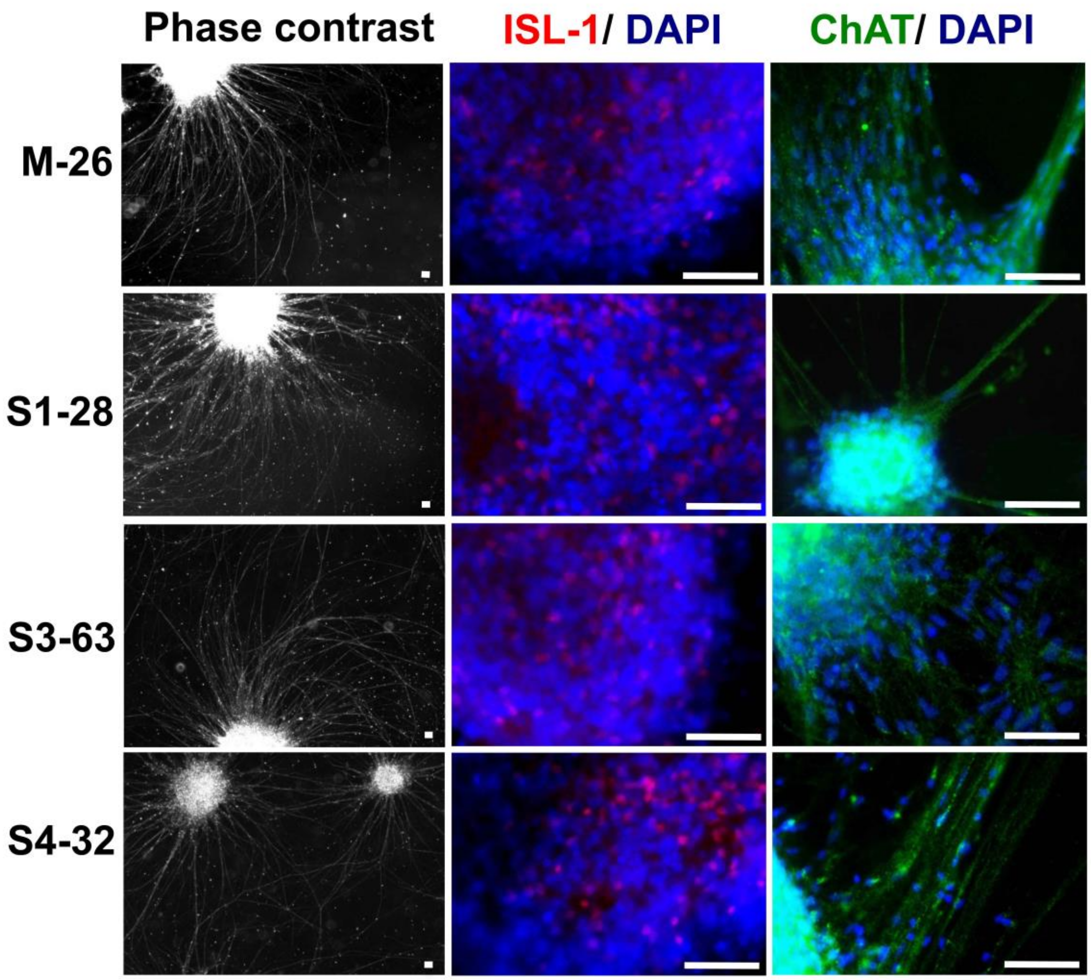

Supplementary Fig. S6. Generation of post-mitotic MNs. Rosettes from selected iPSC clones were grown in suspension as Nsphs for 13 days and plated on poly-L ornithine-laminin coated coverslips for differentiation. Bright field microscopy shows long neurites extending from the clusters of all clones a few days after plating. Immunofluorescence analysis confirmed MN identity by ISL-1 (red) and ChAT (green) expression on days 35 and 46 of MN differentiation, respectively. Nuclei were counterstained with DAPI. The images shown for each clone were taken from different fields/wells. Scale bar = 50 µm. Abbreviations: ISL-1, Insulin gene enhancer protein Islet-1; ChAT, Choline acetyl transferase; DAPI, 4',6-diamidino-2-phenylindole.

Supplementary Figure S7

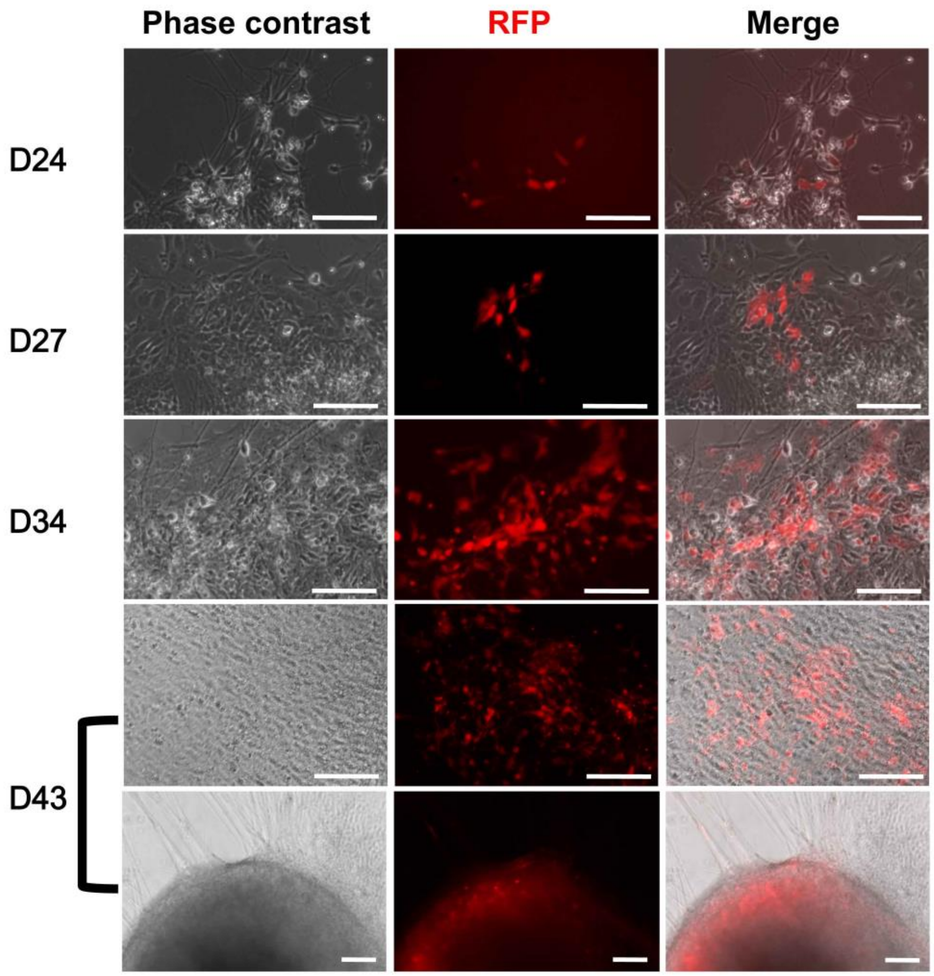

Supplementary Fig. S7. Live follow-up of MN production in differentiating iPSC cultures. D22 Nsphs from iPSC clone S3-63 were transduced with a lentiviral vector expressing *RFP* under the control of the HB9 MN-specific enhancer/promoter. The proportion of RFP-positive cells increased over time through days 24, 27, 34 and 43 of MN differentiation (corresponding to days 2, 5, 12 and 21 post-transduction, respectively). Images on the bottom row show the typical spread of *RFP* expression seen inside the Nsphs. Scale bar = 100 µm. Abbreviations: RFP, red fluorescent protein.

Supplementary Figure S8

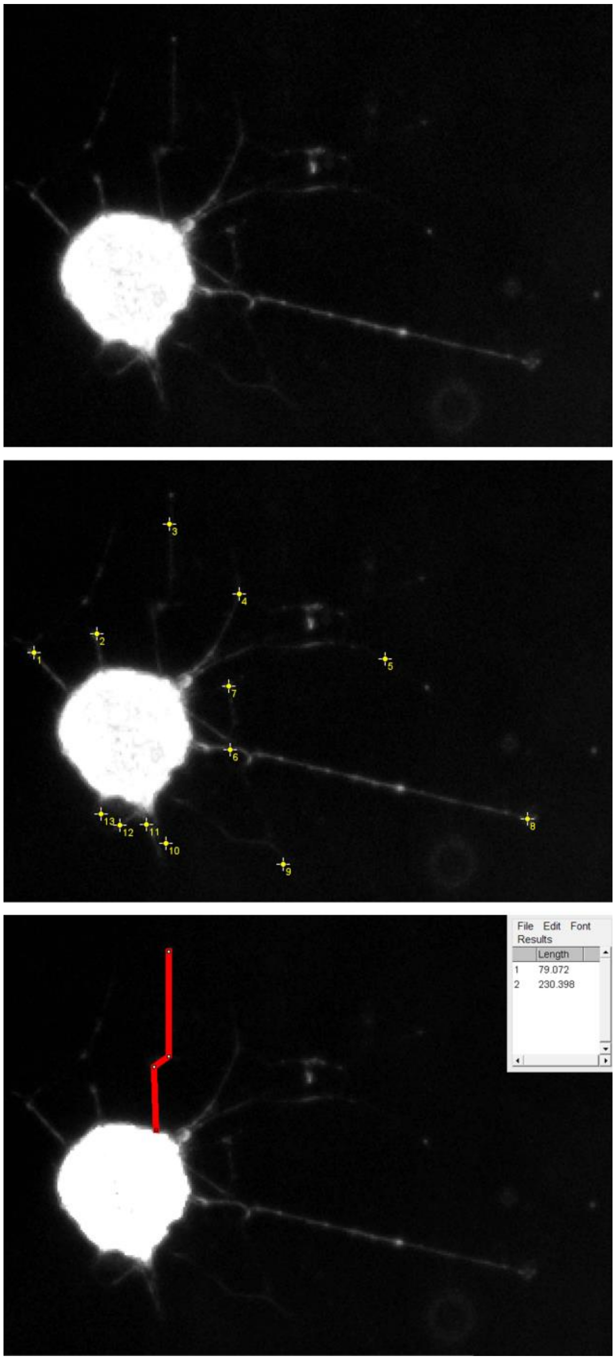

Supplementary Fig. S8. Method employed to perform neurite measurements using ImageJ software. To obtain clearly distinguishable neurites, small differentiating Nsphs were plated sparsely (top). Captured images were analysed to identify and count individual neurites (middle) and obtain length measurements (bottom).

Supplementary Figure S9

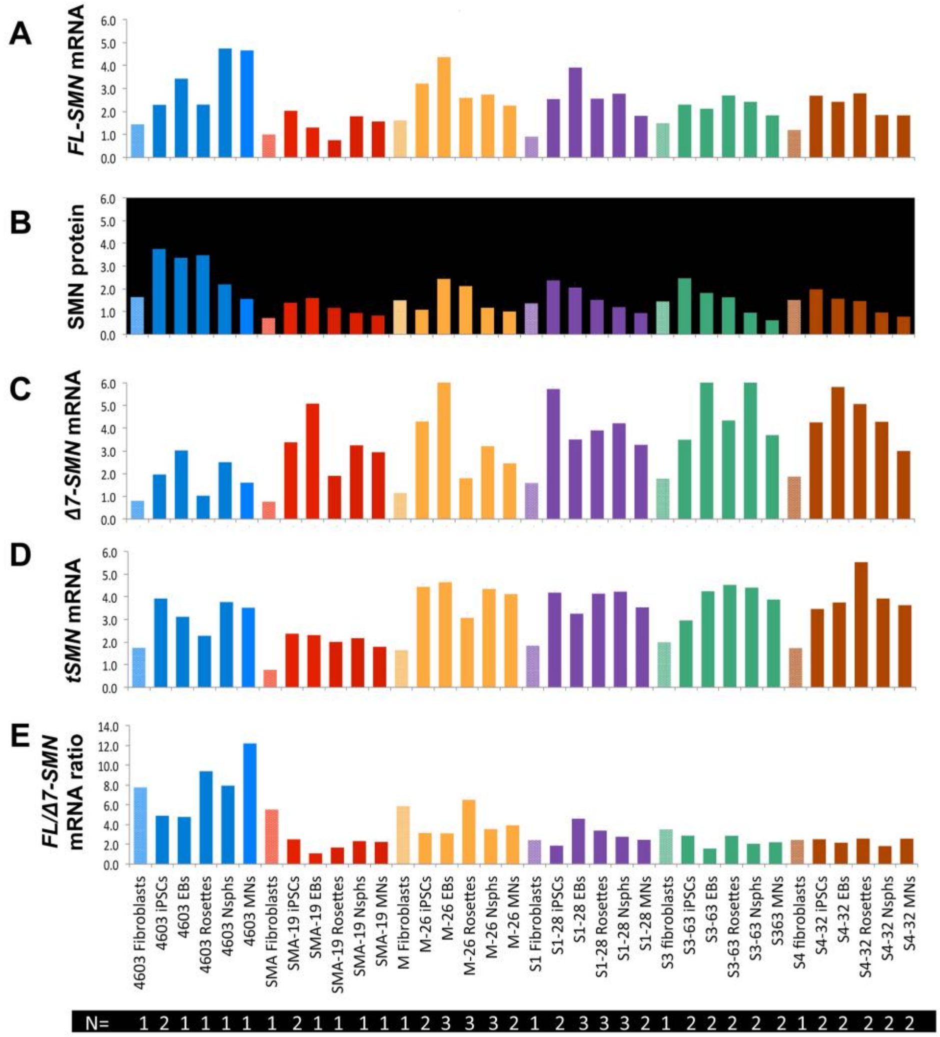

Supplementary Fig. S9. Patient-specific variation of *SMN* mRNA and protein levels during iPSC-MN differentiation. The average levels of *FL-SMN* mRNA, SMN protein, *Δ7-SMN* mRNA, *tSMN* mRNA and *FL/Δ7-SMN* mRNA ratio are shown for all cell types harvested during the iPSC-MN differentiation process of each iPSC clone. Samples were calibrated to a common stock derived from type II SMA fibroblasts. Abbreviations: *tSMN:* total *SMN* transcript; EBs: embryoid bodies; Nsphs: Neuroepithelial-derived spheres; N: number of samples.

Supplementary Figure S10

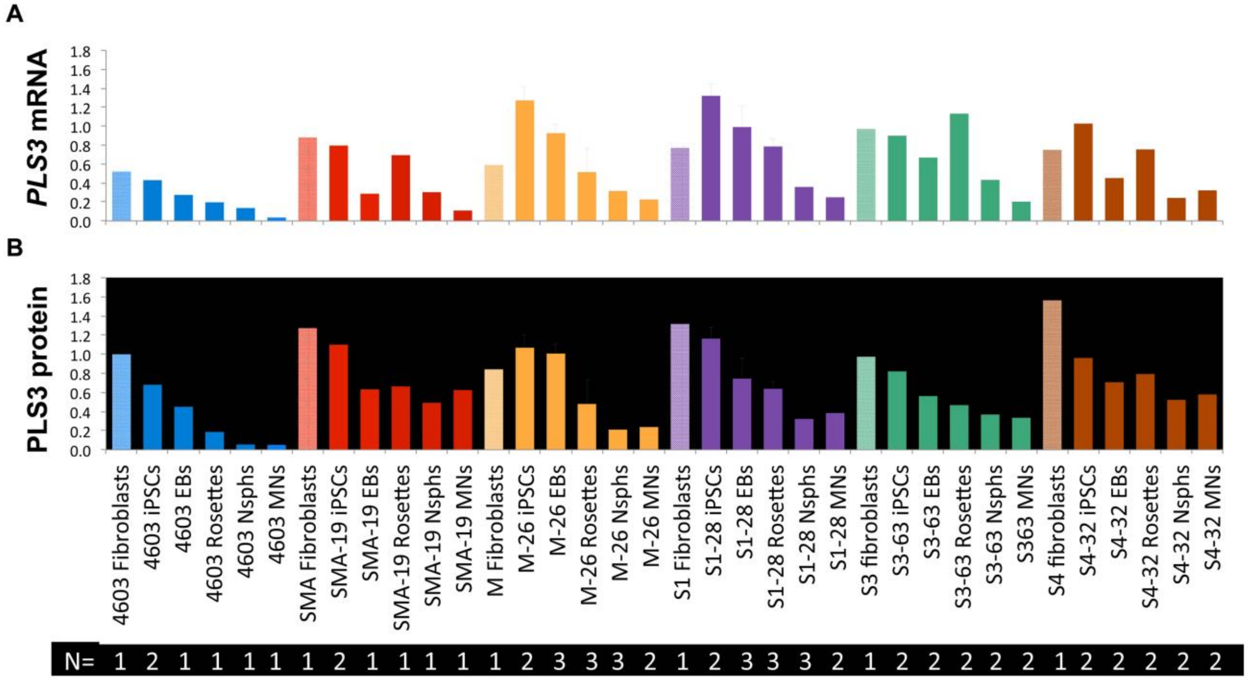

Supplementary Fig. S10. Patient-specific variation of *PLS3* mRNA and protein levels during iPSC-MN differentiation. The average levels of *PLS3* mRNA and protein are shown for all cell types harvested during the iPSC-MN differentiation process of each iPSC clone. Samples were calibrated to a common stock derived from type II SMA fibroblasts. Abbreviations: *tSMN:* total *SMN* transcript; EBs: embryoid bodies; Nsphs: Neuroepithelial-derived spheres; N: number of samples.

Supplementary Figure S11

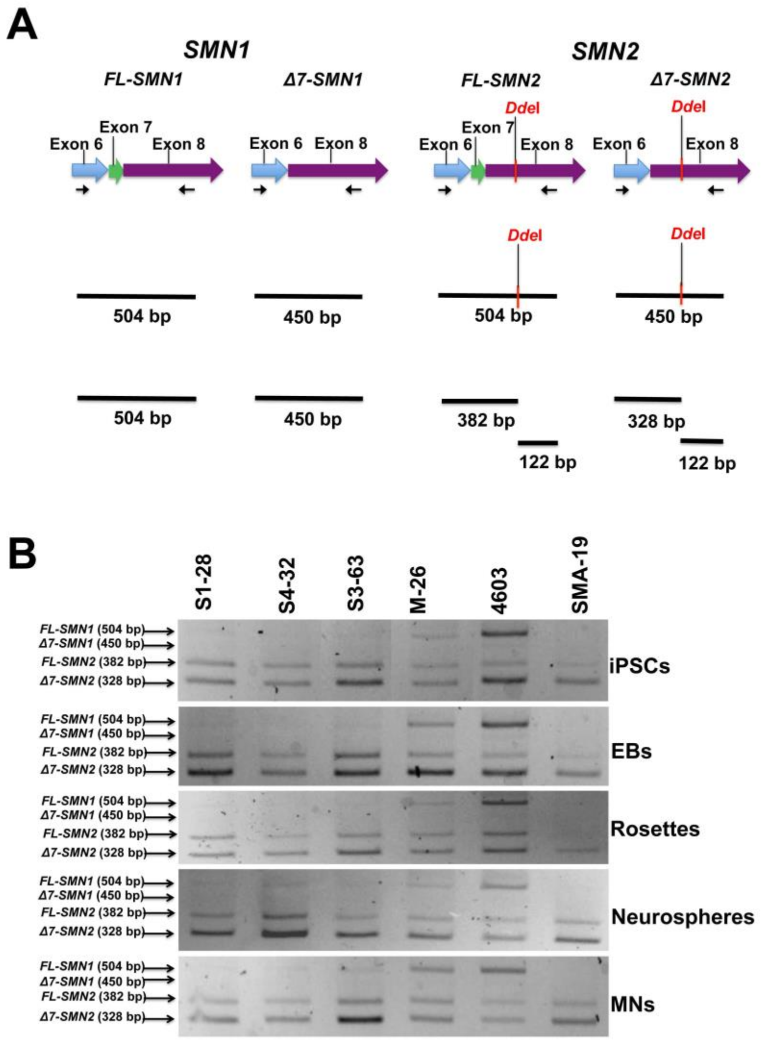

Supplementary Fig. S11. Allelic origin of *SMN* transcripts. (A) Diagram showing the amplification and restriction pattern analysis performed for the determination of the locus from which *FL-SMN* transcripts originate. Top row shows relevant *SMN* transcript regions subjected to amplification, including exons, primers and the diagnostic *Dde*I restriction site present in *SMN2* PCR products. The middle row displays the expected RT-PCR products (504-bp and 450-bp fragments, corresponding to *FL-SMN* and *Δ7-SMN* transcripts respectively). The bottom row indicates expected fragments following *Dde*I digestion of the PCR products (*SMN1* products are not digested, but *FL-SMN2* transcripts generate 382-bp and 122-bp fragments, while *Δ7-SMN2* transcripts produce 328-bp and 122-bp fragments, respectively). (B) *Dde*I restriction analysis of iPSC (D0), EB (D7), rosette (D15), Nsph (D28) and MN (D42) amplification products from the six clones under study. Abbreviations: EB, embryoid bodies; Nsph: Neuroepithelial-derived spheres.
